# Subjective estimates of uncertainty during gambling and impulsivity after subthalamic deep brain stimulation for Parkinson’s disease

**DOI:** 10.1038/s41598-019-51164-2

**Published:** 2019-10-15

**Authors:** Saee Paliwal, Philip E. Mosley, Michael Breakspear, Terry Coyne, Peter Silburn, Eduardo Aponte, Christoph Mathys, Klaas E. Stephan

**Affiliations:** 1 Translational Neuromodeling Unit (TNU), Institute for Biomedical Engineering, University of Zürich and Swiss Federal Institute of Technology (ETH Zürich), Zürich, Switzerland; 2 Systems Neuroscience Group, QIMR Berghofer Medical Research Institute, Herston, Queensland Australia; 3 Neurosciences Queensland, St Andrew’s War Memorial Hospital, Spring Hill, Queensland Australia; 4 Queensland Brain Institute, University of Queensland, St Lucia, Queensland Australia; 5 Faculty of Medicine, University of Queensland, Herston, Queensland Australia; 6 Brizbrain and Spine, the Wesley Hospital, Auchenflower, Queensland Australia; 7 Max Planck Institute for Metabolism Research, Cologne, Germany; 8 Wellcome Trust Centre for Neuroimaging, University College London, London, UK; 9 Scuola Internazionale Superiore di Studi Avanzati (SISSA), Trieste, Italy

**Keywords:** Learning algorithms, Predictive markers, Translational research, Parkinson's disease

## Abstract

Subthalamic deep brain stimulation (DBS) for Parkinson’s disease (PD) may modulate chronometric and instrumental aspects of choice behaviour, including motor inhibition, decisional slowing, and value sensitivity. However, it is not well known whether subthalamic DBS affects more complex aspects of decision-making, such as the influence of subjective estimates of uncertainty on choices. In this study, 38 participants with PD played a virtual casino prior to subthalamic DBS (whilst ‘on’ medication) and again, 3-months postoperatively (whilst ‘on’ stimulation). At the group level, there was a small but statistically significant decrease in impulsivity postoperatively, as quantified by the Barratt Impulsiveness Scale (BIS). The gambling behaviour of participants (bet increases, slot machine switches and double or nothing gambles) was associated with this self-reported measure of impulsivity. However, there was a large variance in outcome amongst participants, and we were interested in whether individual differences in subjective estimates of uncertainty (specifically, volatility) were related to differences in pre- and postoperative impulsivity. To examine these individual differences, we fit a computational model (the Hierarchical Gaussian Filter, HGF), to choices made during slot machine game play as well as a simpler reinforcement learning model based on the Rescorla-Wagner formalism. The HGF was superior in accounting for the behaviour of our participants, suggesting that participants incorporated beliefs about environmental uncertainty when updating their beliefs about gambling outcome and translating these beliefs into action. A specific aspect of subjective uncertainty, the participant’s estimate of the tendency of the slot machine’s winning probability to change (volatility), increased subsequent to DBS. Additionally, the decision temperature of the response model decreased post-operatively, implying greater stochasticity in the belief-to-choice mapping of participants. Model parameter estimates were significantly associated with impulsivity; specifically, increased uncertainty was related to increased postoperative impulsivity. Moreover, changes in these parameter estimates were significantly associated with the maximum post-operative change in impulsivity over a six month follow up period. Our findings suggest that impulsivity in PD patients may be influenced by subjective estimates of uncertainty (environmental volatility) and implicate a role for the subthalamic nucleus in the modulation of outcome certainty. Furthermore, our work outlines a possible approach to characterising those persons who become more impulsive after subthalamic DBS, an intervention in which non-motor outcomes can be highly variable.

## Introduction

The subthalamic nucleus (STN) is a subcortical nucleus of central pathophysiological relevance for Parkinson’s disease (PD). In PD, STN neurons display abnormal patterns of burst firing^1^ and low-frequency synchronisation of local field potentials^2^. By modulating this pathological network activity, subthalamic deep brain stimulation (DBS) for PD improves motor symptoms such as bradykinesia, tremor and rigidity^3^. However, STN-DBS has also been linked to neuropsychiatric symptoms, particularly impulsivity^4,5^, an issue of substantial clinical importance. The STN is a second input station to the basal ganglia, receiving direct cortical projections from the frontal lobe (‘hyperdirect’ pathway)^6^. This route permits basal ganglia inhibitory tone to be directly modulated by cortical regions. Functional and structural brain imaging support the role of this pathway in motor inhibition^7,8^. Following STN-DBS, participants with PD make commission errors (errors in which participants execute an erroneous action)^9^ and take longer to cancel an action^10^, suggesting an impairment in action restraint. Errors in the Stroop^11^ and random number generation tasks^12^ suggest increased sensitivity to cognitive interference and impaired task-switching. When faced with decisional conflict, patients with subthalamic DBS speed up rather than slow down their decision-making^13,14^. These findings suggest a role for the STN in ‘braking’ cognitive-associative circuits in the basal ganglia. It is not clear, however, whether such chronometric aspects of decision-making are sufficient to explain the complex picture of impulsivity after STN-DBS^15^. For example, subthalamic DBS may modulate contingencies in reinforcement learning^16,17^, suggesting a role for the STN in valuation.

A potential computational mechanism underlying impulsivity is the estimation of uncertainty. If the longer-term outcomes of actions are not (or do not seem to be) predictable, prospective thinking may be replaced by seeking immediately available outcomes – a policy that would manifest behaviourally as impulsivity^18^. More specifically, subjective uncertainty about environmental dynamics, or the longer-term consequences of actions, has been associated with a tendency to reduce reflection and long-term planning, and favour short-term over long-term outcomes^19–21^. For example, consider deciding how to invest a sum of money in order to maximise profit. In a dependable economic setting with a reliable government, low unemployment, steadily rising house prices and a stable stock market, one might be confident that a long-term investment in property, business or shares would pay off highly. However, if the long-term outlook was unpredictable, with the possibility of a military coup, high unemployment, crashing property market and volatile stocks, one might decide to spend the money now and enjoy its worth in the short-term. This connection between uncertainty and impulsivity is also seen in PD. PD participants with impulse control disorders (ICDs) ‘jump to conclusions’ in an information collection task (the beads task) more quickly than PD participants without ICDs, a finding that relates informational uncertainty to impulsivity^22^. A computational modelling study of behaviour across three tasks commonly used to probe impulsivity (information sampling, temporal discounting and novelty bias) suggested that PD participants with ICDs are more uncertain about the relationship between possible actions and future rewards than patients without ICDs^23^. Based on these findings, we hypothesised that changes in impulsivity after STN-DBS may also relate to estimates of environmental uncertainty about future rewards.

A paradigmatic approach to inference and learning under uncertainty uses Bayes’ theorem to understand how prior knowledge (represented as a probability distribution known as the *prior*) is combined with new information from the environment (the *likelihood*) in order to update beliefs (the *posterior*). To obtain the posterior, a Bayesian agent inverts a ‘generative’ model (that describes how noisy sensory data result from environmental states); this corresponds to perception. Inferring environmental states from noisy sensory data allows the agent to plan actions that take into account the uncertainty of the environment^24^. Human behaviour often closely resembles those of Bayesian agents, for example, during low level sensory processing^25,26^, sensorimotor learning^27,28^, and higher-level reasoning^29^, although approximations to ideal Bayesian inference are likely required for most domains of cognition^30–32^.

Critically, a Bayesian perspective can accommodate multiple forms of uncertainty, beyond sensory noise. For example, the agent’s environment might change over time. In order to account for this environmental uncertainty (or volatility), Bayesian agents are able to modulate the rate at which they learn (update their beliefs). This learning rate can be linked to an agent’s encoding of volatility^33–35^. For instance, in more volatile environments, estimates of uncertainty (and thus learning rate) should be higher so that more emphasis is given to very recent information; at the same time, predicting the longer-term consequences of actions becomes more difficult. Additionally, uncertainty around how to best translate an uncertain belief about the environment into the selection of an action that will eventually lead to a reward is yet another source of noise that could contribute to observed stochasticity in behaviour. This link between uncertainty and decision-making may be of crucial importance for impulsivity^23^. Furthermore, individual differences in approximate Bayesian inference plausibly contribute to inter-individual variability in behaviour. Such differences can be quantified using models with subject-specific parameters concerning, for instance, the estimation of environmental volatility^36^ or the formation of unusually confident or ‘precise’ beliefs^37^.

In prior work, we found that subject-specific parameter estimates encoding different aspects of uncertainty (such as an estimate of environmental uncertainty, the tonic volatility *ω*) related to a clinical measure of impulsivity in a cohort of healthy subjects^19^. This finding supported a mechanistic understanding of impulsivity from the perspective of uncertainty. Based on these results, we sought to assess whether similar relationships between computational measures of uncertainty and impulsivity were observed in a population of persons with PD undertaking subthalamic DBS. Moreover, given the central role of this brain region in decision-making, we investigated whether receiving subthalamic DBS was associated with changes in the encoding of uncertainty that were connected to increases in impulsivity in certain persons. Finally, we assessed whether a perioperative computational characterisation of subjective aspects of uncertainty (estimates of environmental volatility and stochasticity of belief-response mappings) would be associated with longitudinal changes in impulsivity during clinical follow up. This latter question is clinically important as the non-motor outcomes from subthalamic DBS can be varied. Some centres advocate for the use of DBS to address impulsivity and compulsivity amongst persons with PD on dopamine agonist medication, as subthalamic DBS allows these agents to be substantially reduced or even withdrawn^38^. However, other centres report the emergence of harmful impulsivity subsequent to DBS, in persons with no prior history of clinically-significant psychiatric symptoms^39–43^. At present, there is little evidence to guide the identification of surgical candidates at risk of postoperative impulsivity^4,44^.

In this analysis, we employed a similar computational framework to that previously reported^19^, applying a hierarchical Bayesian model (the Hierarchical Gaussian Filter, HGF) to behavioural data from 38 participants with PD who played a virtual casino before and after subthalamic DBS. By allowing participants to vary their bet size, switch between slot machines and place ‘double or nothing’ bets, we could estimate how participants not only inferred the trial-by-trial probability of winning, but also updated higher-order beliefs about the fluctuations (volatility) of a slot machine’s winning probability. Similar to the rationale outlined in prior work^19^, we believe that a naturalistic paradigm engenders increased behavioural engagement, allowing us to quantify behaviour that has a higher fidelity to ‘real world’ impulsivity. Additionally, model-based estimates derived from the computational framework may afford us an individual profile of how each participant represented (and responded to) environmental uncertainty. We assessed these findings against standard measures of impulsivity derived from clinical assessment and questionnaires, focusing our attention on self-reported impulsivity as measured by the Barratt Impulsiveness Scale (BIS). Our computational analysis examined how DBS changes the manner in which persons with PD engage in Bayesian belief updating, and whether changes in subject-specific estimates of uncertainty before and after DBS relate to changes in impulsivity.

## Materials and Methods

### Subjects

Prior to the commencement of data collection, the full protocol was approved by the Human Research Ethics Committees of the Royal Brisbane & Women’s Hospital, the University of Queensland, the QIMR Berghofer Medical Research Institute and UnitingCare Health. All research was performed in accordance with relevant guidelines and regulations. All participants gave written, informed consent to participate in the study.

Thirty-eight participants with PD undertaking STN-DBS were consecutively recruited at the Asia-Pacific Centre for Neuromodulation in Brisbane, Australia. All participants met the UK Brain Bank criteria for PD^45^. No participants met the Movement Disorder Society criteria for dementia^46^. The PD subtype and the Hoehn and Yahr stage at device implantation was recorded^47^. Patients underwent bilateral implantation of Medtronic 3389 or Boston Vercise electrodes in a single-stage procedure. Stimulation was commenced immediately using microelectrode recording data to identify the optimal contact. Further contact testing took place over the following week as an inpatient, with participants returning to the DBS centre following discharge for further stimulation titration, guided by residual motor symptoms. Further details have previously been reported^48,49^.

### Neuropsychiatric assessment

Impulsivity amongst participants was assessed with patient and clinician-rated instruments prior to STN-DBS and subsequently 2 weeks, 6 weeks, 13 weeks and 26 weeks postoperatively (see Supplementary Fig. 1 for a study flowchart). A range of measures were obtained, to account for the fact that impulsivity is not a unitary construct. These included the Barratt Impulsiveness Scale 11 (BIS) and second-order factors attentional, motor and non-planning^18^; the Questionnaire for Impulsive-Compulsive disorders in PD Rating Scale (QUIP-RS)^50^; the delay discounting task^51^; the Excluded letter fluency task (ELF)^52^; and the Hayling test^53^. Further information on these instruments is detailed in the Supplementary Information. Additional neuropsychiatric symptoms were captured with the Beck Depression Inventory II (BDI)^54^; the Empathy Quotient (EQ)^55^; the Geriatric anxiety inventory (GAI)^56^; and the Apathy Scale^57^. For each self-report scale, participants were instructed to refer to ‘the last two weeks’, in order to obtain a measurement of current ‘state’. At each visit, PD motor symptoms were assessed using the UPDRS Part III motor examination^58^. Dopaminergic medication was recorded and converted to a levodopa-equivalent daily dose (LEDD) value^59^.

### Design and setting

Participants completed the experimental task prior to DBS and at 13-weeks post-DBS. Participants were ‘on’ medication and stimulation for all assessments. We opted against a counterbalanced ‘off’ and ‘on’ DBS assessment at the same visit for several reasons. First, our aim was to provide a naturalistic insight into the subtle behavioural changes that emerge as patients transition from dopaminergic therapies to subthalamic stimulation; changes in levodopa equivalent daily dose were included as co-variates in our analyses. Second, our experience is that many patients would not tolerate the DBS ‘off’ state without severe discomfort. Thirdly, despite allowing DBS washout, plastic network effects of chronic DBS may persist and contaminate findings in an on-off design.

### Task

**Figure 1:**
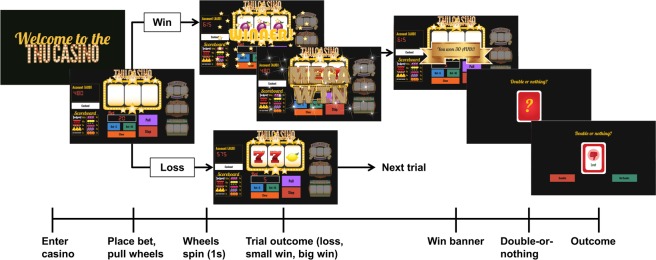
Slot machine gambling paradigm: The task consists of 100 trials. On every trial, players are able to place a bet of unlimited magnitude, switch slot machines or ‘cash out’, exiting the casino and returning again on another virtual ‘day’. The overall win probability is 25%, with wins split into big wins and small wins. The two possible types of losses are near-misses, in which the first two wheels are the same and the third is different (i.e. AAB) or a true loss, in which all the wheels are different (i.e. ABC). Game play proceeds as follows. Each trial begins with the slot machine main screen loading, displaying the player’s account value. The player then places a continuous-valued bet amount, incremented in units of 5 or 10 AUD. After the player has placed a bet, he or she presses the ‘Pull’ button and watches as the wheels begin to spin. At any point, the player has the ability to press the ‘Stop’ button, ending the trial and subsequently revealing the outcome of the three wheels. Unbeknownst to the participant, pressing the stop button has no effect on the trial outcome. If the stop button is not pressed, the trial times out after 5 seconds, and the player sees the outcome of the first, second and third wheel sequentially. On trials in which the outcome is a win, there are ten possible reward grades (or multiples of the bet amount). After every win trial, players are offered a possible ‘double-up’ option, during which players are given 3 seconds to decide whether or not to engage in a ‘double-or-nothing’ option, thereby risking his or her entire win amount. If the player elects to engage in this gamble, a card flips over revealing the result, and subjects are taken to the next trial. If the player does nothing, or decides not to gamble, he or she is taken to the next trial. For each loss trial, players are taken directly to the beginning of the next trial. Again, the trajectory of win-loss outcomes is fixed, ensuring comparable inference upon perceptual and response parameters across participants.

We employed a modified version of an established slot machine gambling paradigm validated in healthy controls^19^. Subjects read an instruction screen and played through 5 training trials, after which they entered a ‘virtual’ casino, starting with 2000 AUD available to gamble and playing 100 trials (Fig. 1). The win-loss likelihood of the slot machines was predetermined and changed at regular intervals. On completion of the task, participants received a small monetary reward proportional to their total winnings. The naturalistic gambling task allows for risk-taking and impulsive behaviour to be expressed and offers four actions on each trial, each of which reflect exploration, and thereby, risk-taking.

(i) Bet Increase: increasing the amount wagered on consecutive trials (minimum of 5 AUD per bet, no maximum)
(ii) Machine-Switch: switching between slot machines (four machines in total)
(iii) Casino Switch: cashing out and switching ‘virtual’ casino days
(iv) Double-Up: engaging in a secondary double-or-nothing gamble on all win trials

As in our previous work^19^, these responses, together with trial-wise outcome information (wins/losses), served as the input for our computational models (for a brief summary, see below). Details on the paradigm and computational modelling can be found in prior work^19^ and the Supplementary Material.

### Computational modelling

#### The hierarchical gaussian filter (HGF)

**Figure 2:**
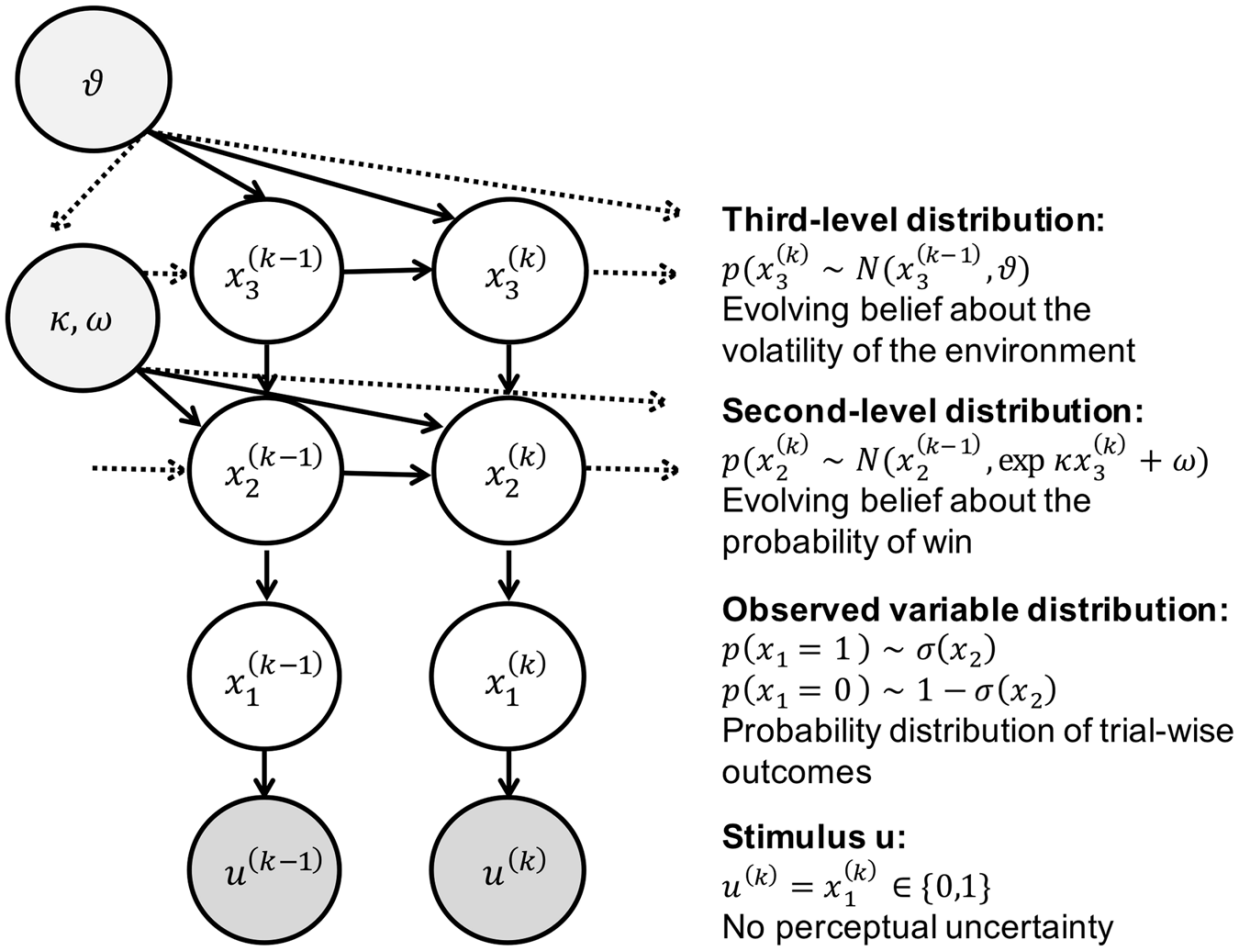
The Hierarchical Gaussian Filter (HGF): *u*^(*k*)^ represents binary observations (true wins = 1, and losses = 0, in the case of the slot machine). Binary inputs are represented on the first level, ${x}_{1}^{(k)}$ via a Bernoulli distribution, around the probability of win or loss, ${x}_{2}^{(k)}$. In turn, ${x}_{2}^{(k)}$ is modelled as a Gaussian random walk, whose step-size is governed by a combination of ${x}_{3}^{(k)}$, via coupling parameter *κ*, and a tonic volatility parameter *ω*. ${x}_{3}^{(k)}$ also evolves as a Gaussian random walk over trials, with step size *ϑ* (meta-volatility). In this investigation, after observing trial-wise outcomes (win or lose), the gambler updates her belief about the probability of win on a given trial *k* $({x}_{2}^{(k)})$, as well as how swiftly that slot machine is moving between being ‘hot’ (high probability of win) or ‘cold’ (low probability of win) ${x}_{3}^{(k)}$. On any trial, the ensuing beliefs then provide a basis for the gambler’s response, which may be to increase the bet size, ‘double up’ after a win, switch to a new slot machine or leave the casino.

The HGF is a hierarchical Bayesian model^34,35^ (Fig. 2) where each level of the hierarchy encodes distributions of environmental variables (in ascending complexity) that evolve as Gaussian random walks. The HGF is an extension of the model presented in Behrens *et al*.^33^, and describes an agent whose learning rate is a function of his or her uncertainty. In the HGF, an agent is assumed not only to represent current environmental contingencies, but also to track how these contingencies change over time (volatility), and to what degree volatility itself is constant (tonic volatility) or may change in time (phasic volatility). Importantly, the agent modelled in the HGF employs these representations to make predictions about emerging environmental fluctuations and future sensory feedback. Furthermore, the agent is able to encode the precision of each prediction and use these precision estimates to scale trial-wise updates of beliefs about the environment and its statistical structure. Each level of the HGF is coupled such that higher states determine how quickly the next lower state evolves, with the lowest hierarchical level representing sensory events.

Inversion of this ‘perceptual model’ produces subject-specific parameter estimates that determine the nature of the coupling between levels of the HGF. Inverting this model under generic (mean-field) approximations results in analytical belief-update equations, in which trial-wise belief updates are proportional to prediction errors (PEs) weighted by uncertainty (or its inverse, precision). The subject-specific parameters shape an individual’s approximation to ideal Bayesian inference, specifically how phasic and tonic volatility impacts trial-wise estimates of uncertainty at all levels of the hierarchy. Posterior estimates of HGF parameters can thus be regarded as a compact summary of an individual’s uncertainty processing during an experiment.

Furthermore, in a ‘response model’, trial-wise beliefs are probabilistically linked to observed trial-wise decisions. Inverting both perceptual and response models allows for estimating the parameters; this corresponds to Bayesian inference (of an observer) on Bayesian inference (of an agent)^24^. An informal description is given below and a formal summary is provided in the Supplementary Material.

#### The perceptual model

The HGF is used to infer how an individual subject learns about hierarchically-coupled environmental quantities under different forms of uncertainty (including volatility). In our case, the lowest level of the HGF, *x*_1_, represents the trial-wise binary outcome (win or loss) in the slot machine. This derives from a sigmoid transformation of *x*_2_ representing winning probability in logit space (i.e., whether the machine is currently ‘hot’ or ‘cold’ and likely (or not) to pay out). *x*_2_ evolves as a Gaussian random walk whose step size is a function *f*_2_(*x*_3_) of a third-level variable, *x*_3_, which performs a Gaussian random walk of its own. *x*_3_ represents the slot machine’s ‘volatility’, the speed at which it fluctuates between ‘hot’ and ‘cold’ states. The coupling function *f*_2_ between levels, contains subject-specific parameters *κ* and *ω* that determine an individual’s approximation to ideal Bayesian inference. Finally, the parameter *ϑ* at the highest level denotes how quickly volatility itself is changing (meta-volatility). A detailed derivation of the exact equations can be found in Mathys *et al*.^34^.

More concretely, in the context of our study, parameters *ω* and *ϑ* at the second and third level of the hierarchy, respectively, encode different aspects of subjective estimates of uncertainty. Specifically, these estimates concern environmental uncertainty, i.e., hidden fluctuations (volatility) of environmental states (for details, see Mathys *et al*.)^34^. These volatility estimates are potentially important for explaining the observed behaviour because they shape participants’ belief updates about the slot machine and their ensuing choices about gambling. Parameter *ω* represents a subject’s estimate of tonic volatility, i.e., how quickly a slot machine could be moving from a state where it is likely to pay out (running ‘hot’) to a state where it is not (running ‘cold’) and vice versa. Parameter *ϑ* encodes a subject’s estimate of meta-volatility, i.e., the tendency of volatility itself to change over time. Larger values of each parameter correspond to greater uncertainty in the subject’s perceptual inference process.

#### The response model

The response model maps a subject’s beliefs (obtained by inverting the perceptual model under given parameter values) to observed gambling behaviour. Here, we use a sigmoidal response model^34^; if this function is steep, there is a close relationship between current perceptual beliefs and betting behaviour. Conversely, a gentler sigmoidal slope results in a more stochastic mapping of beliefs to behaviour. This response function has a parameter, *β*, the decision ‘temperature’ (also known as the inverse temperature), that determines the steepness of the sigmoid and thus the degree of stochasticity in the belief-to-choice mapping. The larger the value of *β*, the steeper the function, and the more deterministic is the relationship between a subject’s belief and their actions. In this paper, we test the following two variants of this response model:

(i) ‘Standard’ HGF: *β* = *constant*, i.e., the mapping from beliefs to behaviour is fixed across the experiment. This parameter is estimated for each subject.
(ii) ‘Uncertainty-driven’ HGF: $\beta =1/{\sigma }_{2}^{(k)}$, where ${\sigma }_{2}^{(k)}$ is the variance of the inferred probability of win on trial *k*. That is, the response behaviour dynamically adapts to the precision of the subjects’ belief about the current probability of winning.

#### Perceptual variable

Based on previous work that examined different computational models of our slot machine paradigm^19^, the perceptual variable used here was simple: a binary variable in which wins were represented by 1 and losses by 0. This binary representation of win or loss in the task allows for increased interpretability of model parameters in measuring uncertainty-updating and impulsive-responding in reaction to a binary win/loss outcome.

#### Response variable

The response variable is a binary representation of actions associated with risk taking. It is constructed using a logical OR operator on four choices during the slot machine paradigm: bet increases, machine switches, double-ups and casino switches. For each trial, the response variable takes a 1 when any of these four events occur, and 0 otherwise. For details, please see Supplementary Table 1.

While these actions might at first glance appear to relate to different behaviours, they all share a common theme in that they enhance outcome variance and thus the amount of risk the player takes in the game. For example, increasing bet size from one trial to the next results in higher reward variability in the trial outcome, thereby making the player more susceptible to larger wins and losses. In aggregate, these four actions relate to a player’s risk-taking tendencies (described further in Supplementary Section 1.4).

#### Reinforcement learning

As an alternative model, we used a classical associative learning model, Rescorla-Wagner (RW), often used in reinforcement learning (RL)^60^. The RW model updates the probability of a win on trial *k* by combining the probability on trial *k*−1 with a PE weighted by a constant learning-rate. Hence, in contrast to the HGF, the RW model does not have a dynamic learning rate over trials, nor can it account for different forms of perceptual uncertainty—essentially, the RW model corresponds to an HGF with a fixed learning rate. Here, we combine the RW learning rule with the same sigmoidal response model described above, with free parameter *β*, that we estimate on a subject-specific basis. This results in a model that is (i) structurally not dissimilar but less complex than the HGF and (ii) almost identical to the RL model used in a prior investigation of learning after STN-DBS^17^.

#### Model inversion

The HGF and RW models were inverted using population Markov-Chain Monte Carlo (MCMC) sampling^61^. Parameter estimation in the HGF is classically ‘fully Bayesian’ and requires a selection of priors, which influence parameter estimation to a lesser or greater degree. In order to minimise this influence, we used a novel empirical Bayesian inference scheme for the HGF where a Gaussian group-level distribution of parameters is constructed from samples across the group. This group-level empirical prior is then used to obtain posterior parameter estimates in each subject (Supplementary Fig. 2). Subject-specific point estimates for model parameters are calculated as the median value of the subject’s posterior distribution.

Given the clinical constraints of our investigation (to reduce any burden on the participants, we only used 100 trials per episode of gambling, i.e., only half as many as in our previous work)^19^, it was important to ensure that our parameter estimates were robust. Therefore, in order to verify that HGF parameter estimates reliably reflected subject-specific characteristics of uncertainty encoding and decision noise, we tested our ability to recover ground-truth parameter values from simulated response data. In order to assess parameter recoverability, we used three parameter values per parameter (shown in Supplementary Fig. 4) and generated a batch of 38 synthetic response variables based on these assigned values, using the underlying trace of the slot machine as the perceptual variable. We then inverted the HGF and explored the relationship of the recovered parameter estimates, using the median of the posterior, with the ground truth values. When estimating *ω* and *ϑ*, *β* was held fixed; conversely, when estimating for *β*, *ω* and *ϑ* were fixed. This process was repeated for 10 batches across each parameter.

#### Model comparison

As described above, we considered two competing hypotheses of how subjects might incorporate uncertainty into their choice of actions, i.e., two different belief-to-choice mappings in the response model for the HGF (the ‘Standard’ and ‘Uncertainty-driven’ models). These two versions of the HGF were compared with the RW model. As we were primarily interested in the pre-DBS to post-DBS change, we selected the winning model for the pre-DBS measurements. We then evaluated if the parameter estimates of that winning model changed postoperatively. Estimates of the negative free energy (log model evidence) were computed using thermodynamic integration^61^. The negative free energy balances goodness of fit with a complexity penalty. Group-level free energy estimates were compared to select a winning model.

### Data analysis

#### General considerations

All computational modelling and model inversion was performed using MATLAB (Mathworks), employing custom scripts developed from the HGF toolbox version 3 in the open source software TAPAS (http://www.translationalneuromodeling.org/tapas/). Multiple regression analyses were performed using the regstats function in the MATLAB Statistics Toolbox. For all analyses involving multiple comparisons, native *p*-values are presented, accompanied by Holm-Bonferroni correction at *α* = 0.05. To test the significance of individual regressors in multiple regression models, post hoc t-tests were performed.

Neuropsychiatric assessment data from baseline, prior to DBS, was compared with data gathered at 13-weeks post-DBS, when the gambling task was repeated. To test for differences in pre-DBS and post-DBS questionnaire scores and model parameter estimates, a paired t-test was employed when the data were normally distributed and the Wilcoxon signed-rank test otherwise, where distribution was assessed using the Lilliefors test. Gambling behaviours (such as bet increases and machine switches) were also compared at both intervals. Gambling behaviours were regressed against clinical measures of impulsivity to determine significant relations. After determining the winning computational model, model parameter estimates were extracted for each participant and regressed against clinical measures of impulsivity to determine significant associations and predictors of postoperative impulsivity. Based on this previous work showing a significant association between BIS scores and both slot machine behaviour and HGF-based estimates of uncertainty encoding^19^, we focused our analyses on the BIS and its subscales. Perceptual model parameters were extracted in log space: *ω* and *β* are naturally estimated in log space, since they are part of exponential terms in their respective equations (see equation 5 in the supplementary material).

From a clinical perspective, we were interested in examining whether changes in the computational characterisation of individual uncertainty estimates pre- to post-DBS were associated with clinically-relevant changes in impulsivity at any time point after DBS. Our strategy to attempt prediction of clinical outcomes follows the ‘generative embedding’ approach, in which individual predictions are not derived from measured data but from parameter estimates obtained by a generative model^62,63^. Importantly, stimulation-dependent changes in impulsivity may evolve in an unpredictable manner subsequent to DBS, related to variations in DBS programming over time (with considerable adjustments to stimulation in the first six postoperative months). Furthermore, the optimal BIS cut-off score for clinically-significant impulsivity varies by age and disease^64^, with only one existing investigation specific to a PD cohort^65^. Therefore, we examined whether individual changes in parameter estimates associated with the maximum postoperative increase in impulsivity, as measured by the BIS, compared to baseline, across six months of longitudinal follow up.

## Results

### Participant characteristics

Participants were a predominantly middle-aged sample, with a bias towards male gender and akinetic-rigid/mixed phenotype over tremor (Table 1). Most participants had bilateral disease with consequent impairment of functioning in their activities of daily living.

**Table 1:** Demographic and clinical characteristics of PD cohort (n = 38).

Categorical Variable	Total (*n* = 38)	
**Gender**	***n***	**% total**
Male	25	65.8
Female	13	34.2
**Clinical Subtype**	***n***	**% total**
Akinetic-Rigid	13	34.2
Mixed	18	47.4
Tremor	7	18.4
**Continuous Variable**	***Mean*** (***SD***), ***Median*** (***Range***)	
Age (Years)	61.9 (±9.3), 65 (35–76)	
Hoehn & Yahr Stage	2.7 (±0.6), 2.5 (1.5–4)	
Years Since Diagnosis	8.5 (±4.6), 7 (2–21)	

### Neuropsychiatric assessment Pre- and Post-DBS

Concerning symptoms of primary interest (Table 2), there was a small but statistically-significant group-level post-DBS decrease in impulsivity, as measured by the BIS Total, compared to baseline. There was also a significant reduction of motor symptoms assessed using the UPDRS Part III Motor Examination, with a corresponding significant reduction in the requirement for dopaminergic therapy (LEDD). There were no statistically-significant changes in other behavioural measures related to impulsivity, including the Hayling test, the Excluded Letter Fluency task and the delay discounting task. Comparable to the BIS, the QUIP-RS total score demonstrated a trend towards a reduction at 13-weeks post-DBS, but this did not reach significance.

**Table 2:** Neuropsychiatric Assessment Data Pre- and Post-DBS.

Behavioural Measure	Pre-DBS	Post-DBS	Max Impairment	Pre- vs. Post-DBS		
Gaussian Distribution	*Mean* (*SD*), *Median* (*Range*)			*t*-*stat*	*p*-*value*	*Adj*. *p*-*value*
BIS	60.2 (±7.4), 60 (44–76)	57.8 (±9.5), 58 (40–75)	2.3 (±6.4), 3 (−14–17)	2.66	0.011^‡^	0.033*
BDI	11.1 (±5.1), 11 (1–22)	8.4 (±6.6), 6 (1–29)	1.3 (±7.1), 0 (−12–18)	2.29	0.028^‡^	0.056
QUIP-RS	21.4 (±15.7), 19 (0–63)	17.2 (±15.8), 14 (0–55)	5.1 (±13.5), 4 (−25–37)	1.78	0.084	0.084
UPDRS Part III Motor	37.7 (±17.0), 37 (10–91)	30.9 (±12.7), 32 (8–60)	N/A	3.47	0.001^‡^	0.004**
LEDD	1032.9 (±599.4), 988 (0–3450)	334.9 (±199.8), 329 (0–825)	N/A	8.59	<0.001^‡^	<0.001***
**Skewed Distribution**	***Mean*** (***SD***), ***Median*** (***Range***)			***Chi*** **-** ***square***	***p*** **-** ***value***	***Adj***. ***p*-*value***
ELF Rule Violations	9.7 (±5.3), 9 (0–24)	8.4 (±5.0), 8 (1–18)	3.0 (±6.5), 1 (−6–19)	20.2	0.12	0.360
Hayling AB Error Score	11.4 (±11.1), 8 (0–38)	9.4 (±11.3), 5 (0–45)	6.2 (±12.0), 6 (−19–30)	23.6	0.13	0.360
Delay Discount K	0.034 (±0.067), 0.016 (0.00016–0.25)	0.036 (±0.049), 0.016 (0.00016–0.25)	0.041 (±0.077), 0.0128 (−0.15–0.25)	12.6	0.18	0.260

^‡^Indicates significant native p-values, before multiple comparison correction.

***p < 0.001, **p < 0.01, *p < 0.05 where p-values are Holm-Bonferroni corrected for multiple comparisons with *α* = 0.05.

Max impairment refers to the maximum impairment for each outcome across all measurement intervals subsequent to DBS.

For symptoms of secondary interest (and subscales), see Supplementary Table 2. There was considerable variance between subjects across assessment scores at each interval and within subjects across the course of longitudinal follow up (Supplementary Fig. 3).

The BIS and the BDI showed a significant positive correlation at each time point (*ρ*_*pre*_ = 0.46, *p* = 0.003; *ρ*_*post*_ = 0.53, *p* < 0.001), and both showed (near-)significant changes from pre- to post-DBS (Table 2). Therefore, to rule out that impulsivity-related findings were driven by changes in depression, the BDI was included as a covariate when regressing behaviour and model parameter estimates against BIS scores. Whilst the LEDD is conceivably related to impulsivity, it did not correlate with the BIS total (*ρ*_*pre*_ = −0.126, *p* = 0.450; *ρ*_*post*_ = −0.042, *p* = 0.799) and was therefore not included in these regression analyses. However, the QUIP and LEDD correlated strongly at both time points (*ρ*_*pre*_ = 0.42, *p* = 0.008); *ρ*_*post*_ = 0.44, *p* = 0.005), with LEDD decreasing significantly post-DBS. There were no significant correlations between LEDD and the other measures of impulsivity (ELF Rule Violations, Hayling AB Error Score and Delay Discount K). Based on previous work using this task and modelling framework^19^, we focused our attention on exploring impulsivity as measured by the BIS.

### Gambling behaviour

#### Gambling behaviour Pre- and Post-DBS

At the group level, there were no significant differences in the behaviour of participants on the slot machine from pre- to post-DBS (Supplementary Table 3). Due to subjects not engaging in the ‘casino switch’ option, this variable was eliminated from regression analyses.

#### Pre-DBS Regression of BIS scores on Gambling Behaviour

We studied the relationship between pre-DBS gambling behaviour and pre-DBS impulsivity as measured by the BIS (Table 3). The BDI was included in this regression in order to control for changes in clinical state attributable to depressive symptoms. The overall preoperative model including the BDI total was significantly associated with the BIS total score [*F*_(4,33)_ = 3.024, *p* = 0.031]. Post-hoc t-tests on task behaviour revealed that no behavioural variable was significantly related to the BIS individually. When subscales of the BIS were examined, gambling behaviour associated significantly with the BIS Attentional subscale [*F*_(4,33)_ = 4.094, *p* = 0.008], where higher bet sizes corresponded to higher attentional impulsivity (*t*_(37)_ = 2.303, *p* = 0.028) (Supplementary Table 4).

**Table 3:** Slot Machine Behaviour.

Slot Machine Behaviour							
Pre-DBS Slot Machine Behaviour and Pre-DBS BIS							
Dependent Variable	Independent Variables (*b*)						
	Bet Size	Machine Switch	Double-up	BDI	*R* ^2^	F-stat	*p*-value
BIS Total	0.034	0.338	−0.077	0.821	0.268	3.024	0.0341*
**Post-DBS Slot Machine Behaviour and Post-DBS BIS**							
BIS Total	0.018	0.128	0.263	0.712	0.374	4.920	0.003**
**Pre-to-Post Slot Machine Behaviour and Max BIS Increase**							
**Dependent Variable**	**Independent Variables** (***b***)						
	**ΔBet Size**	**ΔMachine Switch**	**ΔDouble-up**	**ΔBDI**	***R*** ^**2**^	**F-stat**	***p*** **-value**
Max BIS Increase	0.055^	0.643^	0.042	0.057	0.299	3.516	0.017*

b values are standardized regression coefficients. ***p < 0.001, **p < 0.01, *p < 0.05. ^ Indicates significant t-statistics, Holm-Bonferroni corrected for multiple comparisons with *α* = 0.05. Max BIS increase refers to the maximum postoperative impairment for impulsivity across all measurement intervals post-DBS.

**Table 4:** HGF model parameters, Pre- and Post-DBS.

	Pre-DBS	Post-DBS	t-stat	*p*-value
*ω*	−8.165 (0.207, −8.740–7.843)	−5.491 (0.261, −6.187–4.952)	−61.328	<0.001^***^
*β*	3.279 (2.802, 0.160–10.157)	2.218 (2.525, 0.096–9.721)	2.124	0.04^*^

Group means are reported with standard deviations and range in parentheses. Model parameters are reported in log space.

***p < 0.001, **p < 0.01, *p < 0.05 where p-values are Holm-Bonferroni corrected for multiple comparisons with *α* = 0.05.

#### Post-DBS regression of BIS scores on gambling behaviour

The full model of postoperative gambling behaviour was also significantly associated with BIS total score [*F*_(4,33)_ = 4.920, *p* = 0.003] (Table 3). Again, post-hoc t-tests revealed that no task behaviour was significant on its own. When subscales of the BIS were examined, gambling behaviour correlated significantly with the BIS Attentional subscale [*F*_(4,33)_ = 8.123, *p* < 0.001]. Post-hoc t-tests revealed that higher bet sizes (*t*_(37)_ = 2.604 *p* = 0.014) and more frequent double or nothing gambles (*t*_(37)_ = 2.589 *p* = 0.014) corresponded to higher BIS Attentional scores (Supplementary Table 5).

Post-DBS, higher bets and more frequent machine switches were significantly associated with higher QUIP-RS scores (Supplementary Table 6). No other measures of impulsivity were significantly associated with pre- or post-DBS slot machine activity.

#### Regression of maximum BIS Increase on Pre- to Post-DBS changes in gambling behaviour

The change in gambling behaviours between the pre- and post-DBS time points were significantly associated with the maximum postoperative increase in BIS score. [*F*_(4,33)_ = 3.516, *p* = 0.017] (Table 3). Post-hoc t-tests revealed that the change in bet behaviour significantly was associated with maximum BIS increase (*t*_(37)_ = 2.866, *p* = 0.007). Additionally, the change in machine switch behaviour was also significantly associated with maximum BIS increase (*t*_(37)_ = 2.219, *p* = 0.034). In other words, changes in betting and slot machine switching behaviours after DBS indexed changes in impulsivity as assessed by the BIS.

### Computational modelling

**Figure 3:**
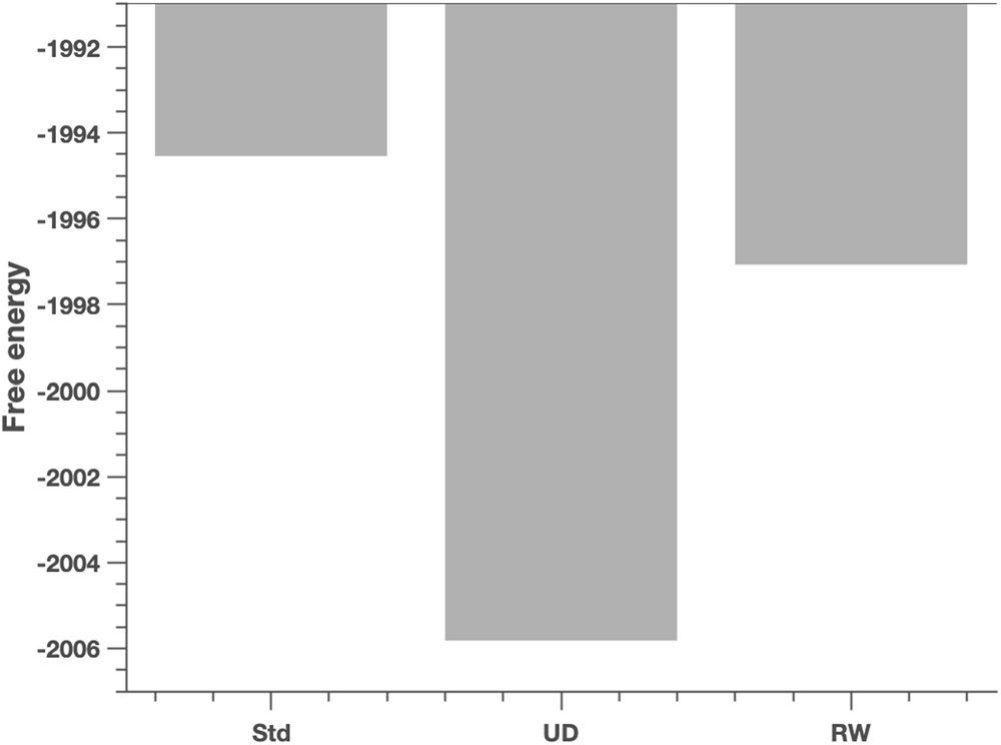
Model Comparison Results: Bayesian model comparison results across the Standard (Std), Uncertainty-driven (UD) and Rescorla Wagner (RW) models, pre- and post-DBS. Shown here are the group-level free energy values for the three models, Std, UD and RW. Pre-DBS model free energies are *F*_*Std*_ = −1994.54, *F*_*UD*_ = −2005.82, and *F*_*RW*_ = −1997.07. The winning model pre-DBS is the standard HGF. The group-level difference in free energy compared to the next best model (the Rescorla-Wagner model) is 2.53, corresponding to a Bayes factor of approximately 12.5.

As described above, we were interested in evaluating the role of uncertainty and its association with postoperative changes in impulsivity in our cohort. We therefore first determined, using Bayesian model comparison, which of our three models best explained pre-DBS behaviour, before evaluating whether the parameter estimates of this winning model changed postoperatively and were associated with postoperative BIS scores. Bayesian model comparison selected the ‘standard’ HGF (with a subject-specific decision temperature in the response model) as the winning model, with a group-level Bayes factor of approximately 12.5, compared to the next best model (the Rescorla-Wagner model) (Fig. 3).

#### Parameter recoverability in the HGF

We tested for parameter recoverability in the HGF, finding that ground truth parameter values for *ω* and *β* could be recovered consistently, but we were unable to reliably recover *ϑ* (for details, see Supplementary Fig. 4). For this reason, we restricted the following analysis to parameters *ω* and *β* when exploring the association between parameter values and questionnaire-based measures of impulsivity.

#### Changes in model parameter estimates Pre- to Post-DBS

**Figure 4:**
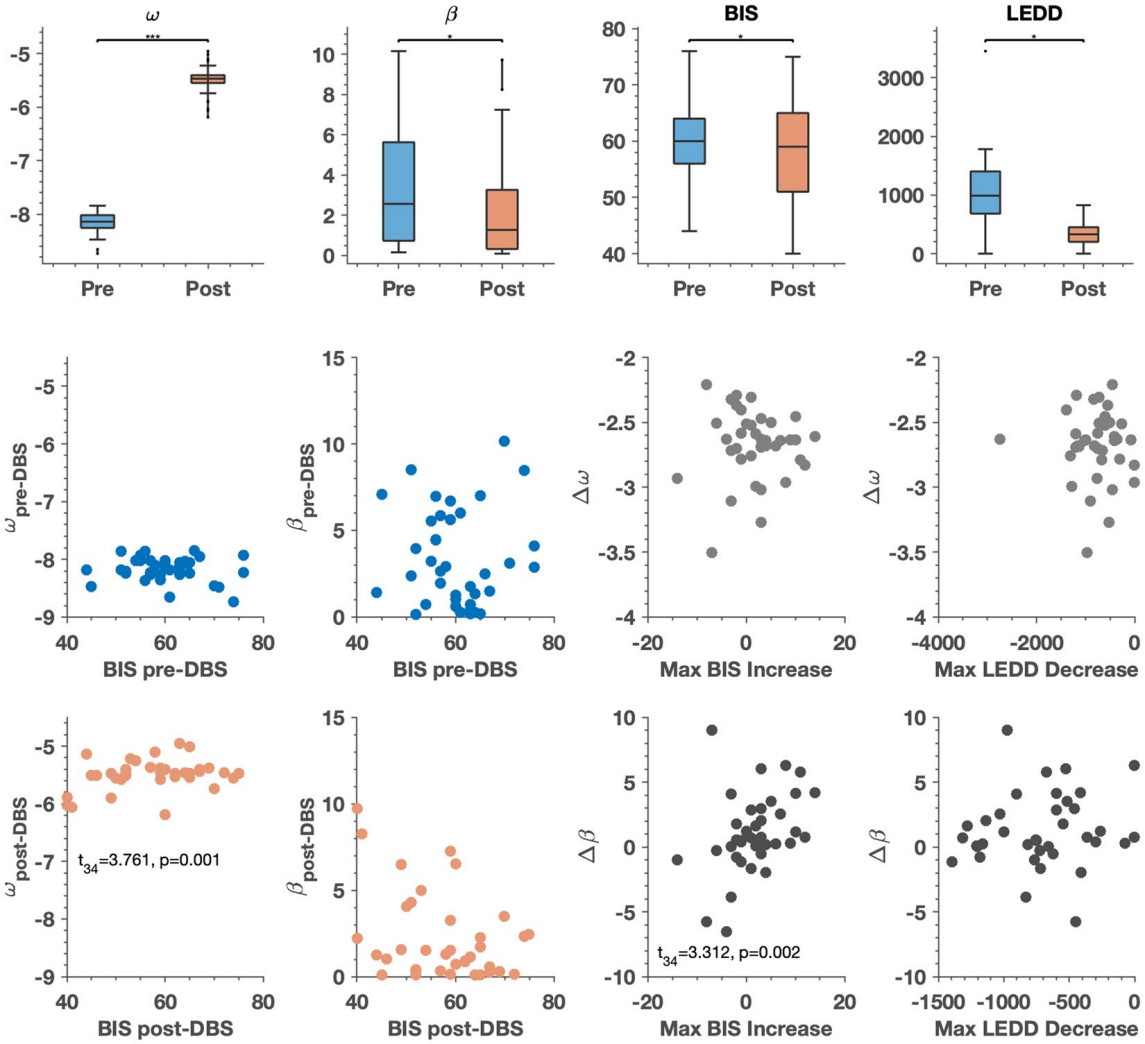
Computational Model Parameters, Pre- and Post-DBS: In the first row of figures, model parameter estimates for *ω* and *β*, BIS scores and LEDD scores are displayed pre- and post-DBS. For the box plots, the central line indicates the median of the distribution, and the top and bottom edges of the box represent the 25^th^ and 75^th^ percentiles respectively. The whiskers extend to the farthest data points that are included in the distribution and are not considered outliers. A paired t-test was performed to determine the pre-DBS vs. post-DBS difference in the distributions of *ω*, *β*, the BIS and the LEDD. Significant differences were observed in estimates of parameter *ω* (*t*_(37)_ = −61.328, *p* < 0.001) and in estimates of parameter *β* (*t*_(37)_ = −2.214, *p* = 0.04) as shown in Table 4. Also shown is the change in BIS pre- and post-DBS (*t*_(37)_ = −2.66, *p* = 0.033), as shown in Table 2. *p*-values are Holm-Bonferroni corrected for multiple comparisons with *α* = 0.05. The second row illustrates the relationship between pre-DBS *ω* and pre-DBS BIS, pre-DBS *β* and pre-DBS BIS and the pre-to-post change in *ω* with the max increase in BIS, as well as the max decrease in LEDD. The third row illustrates the relationship between post-DBS *ω* and post-DBS BIS, post-DBS *β* and post-DBS BIS and the pre-to-post change in *β* with the max increase in BIS. Here, we have removed the outlier in the plot relating the change in *β* to the max decrease in LEDD. These plots serve to better illustrate the results shown in Table 5. Specifically, that greater volatility estimates (*ω*, the tendency of a slot machine’s winning probability to change) were associated with greater maximum postoperative BIS scores, and that greater stochasticity in belief-to-choice mapping (decision temperature - *β*) associated significantly with the maximum postoperative increase in BIS.

Estimates of the HGF model perceptual parameter *ω* significantly increased postoperatively (*t*_37_ = −61.328, p < 0.001), and estimates of *β* significantly decreased (*t*_37_ = 2.124, p = 0.04), implying larger subjective estimates of uncertainty (volatility) and greater stochasticity in the selection of responses after DBS (Table 5 and Fig. 4). *ω* represents a subject’s estimate about the tonic component of environmental volatility; i.e., how quickly the likelihood of winning on a given slot machine might be changing, while *β* represents the decision noise, or the stochasticity involved in the belief-to-choice mapping process.

**Table 5:** Model Parameters.

Model Parameters						
Pre-DBS Model Parameters and Pre-DBS BIS						
Dependent Variable	Independent Variables (*b*)					
	*ω*	*β*	BDI	*R* ^2^	F-stat	p-value
BIS Total	−5.306	−0.370	0.729	0.229	3.372	0.03*
**Post-DBS Model Parameters and Post-DBS BIS**						
BIS Total	17.434^	−0.007	0.943	0.49	10.906	<0.001***
**Max BIS Increase and Change in Model Parameters**						
**Dependent Variable**	**Independent Variables** (***b***)					
	**Δ** ***ω***	**Δ** ***β***	**ΔBDI**	***R*** ^**2**^	**F-stat**	***p*** **-value**
Max BIS Increase	8.681	1.220^	−0.098	0.260	3.987	0.015*
**Max LEDD Reduction and Change in Parameters**						
**Dependent Variable**	**Independent Variables** (***b***)					
	**Δ** ***ω***	**Δ** ***β***		***R*** ^**2**^	**F-stat**	***p*** **-value**
Max LEDD decrease	591.26	89.22^		0.187	4.032	0.02*

b values are standardized regression coefficients. ***p < 0.001, **p < 0.01, *p < 0.05. ^ Indicates significant t-statistics, Holm-Bonferroni corrected for multiple comparisons with *α* = 0.05.

Changes in *ω* and *β* are calculated as the pre-DBS parameter estimate value minus the post-DBS parameter estimate value. Max BIS increase refers to the maximum impairment for impulsivity across all measurement intervals subsequent to DBS. Max LEDD decrease refers to the maximum postoperative reduction of dopaminergic medication across all measurement intervals subsequent to DBS.

#### Pre-DBS regression of BIS scores on model parameter estimates

The full regression model (including the estimates of preoperative perceptual and response parameters *ω* and *β*) was significantly associated with BIS total [*F*_(3,34)_ = 3.372, *p* = 0.03] (Table 5). Post-hoc t-tests on model parameter estimates did not reveal any single parameter to be significantly related to the BIS on its own. When subscales of the BIS were examined, the full regression model associated significantly with the BIS Attentional subscale [*F*_(3,34)_ = 3.314, *p* = 0.031], but again no single parameter was independently significant (Supplementary Table 7).

#### Post-DBS regression of BIS scores on model parameter estimates

The full regression model was significantly associated with BIS total [*F*_(3,34)_ = 10.906, *p* < 0.001] (Table 5). Post-hoc t-tests revealed that *ω* was a significant regressor (*t*_(34)_ = 3.761, *p* = 0.001). The positive regression coefficient for *ω* implies that the greater the subjective estimate of uncertainty (tonic volatility), the higher the BIS score (Fig. 4). In other words, although there was a group level decrease in BIS score pre-post DBS, at an individual level the greater postoperative estimate of uncertainty (i.e., the higher the estimated volatility of the slot machine’s winning probability), the greater the postoperative impulsivity. Thus, for participants with high subjective volatility estimates, there was likely to be a postoperative increase in BIS. When subscales of the BIS were examined, model parameter estimates correlated significantly with the BIS Attentional subscale [*F*_(3,34)_ = 7.777, *p* < 0.001] and the BIS Non-Planning subscale [*F*_(3,34)_ = 7.642, *p* < 0.001]. Post-hoc t-tests revealed that *ω* was also a significant regressor in both of these associations (*t*_(34)_ = 2.874, *p* = 0.007 for BIS Attentional and *t*_(34)_ = 2.561, *p* = 0.015 for BIS Non-Planning) (Supplementary Table 8).

#### Change in model parameters associating with maximum change in BIS

We were interested in whether individual pre- to postoperative changes in estimates of subjective uncertainty would correlate with the maximum postoperative change in impulsivity across six months of follow up post-DBS. The pre-to-post change in model parameter estimates was calculated as the preoperative minus the postoperative parameter estimate. In the case of *ω*, postoperative parameter estimates were significantly higher than preoperative values (in the case of *ω*, higher values imply higher uncertainty), therefore the Δ*ω* value for each subject is negative and implies a post-operative increase in uncertainty. With regards to *β*, postoperative parameter estimate values were significantly lower than preoperative values (in the case of *β*, lower values imply greater stochasticity), therefore the Δ*β* value for most subjects is positive, implying a postoperative increase in the stochasticity of the belief-to-choice mapping. Changes in model parameter estimates were associated with the maximum post-operative increase in the BIS across all longitudinal assessments over six months [*F*_(3,34)_ = 3.987, *p* = 0.015] (Table 5). Post-hoc t-tests revealed that Δ*β* was a significant regressor (*t*_(34)_ = 3.312, *p* = 0.002). The positive regression coefficient here implies that a post-operative increase in decisional randomness related to a greater maximum increase in BIS (Fig. 4).

#### Supplementary analyses

We examined whether dopaminergic medication dosage expressed as a standardised unit (LEDD) was connected to computational model parameters and whether changes in drug doses postoperatively were connected to changes in uncertainty encoding. There was no significant relationship between pre-DBS model parameters and pre-DBS LEDD [*F*_(2,35)_ = 1.008, *p* = 0.375] or post-DBS model parameters and post-DBS LEDD [*F*_(2,35)_ = 0.266, *p* = 0.768]. Additionally, the pre-to-postoperative change in LEDD did not significantly relate to pre-to-postoperative change in BIS (*ρ* = 0.28, *p* = 0.088). Also, the maximum decrease in LEDD across six months of follow up did not significantly relate to the postoperative maximum increase in BIS (*ρ* = 0.29, *p* = 0.073),

However, there was a significant relationship between the change in model parameter estimates and the maximum post-operative decrease in LEDD [*F*_(2,35)_ = 4.032, *p* = 0.027] (Table 5). The BDI was not included in these regression models, as it is not a confound when examining a relationship between model parameter estimates and the LEDD. Post-hoc t-tests showed that Δ*β* was a significant regressor (*t*_(34)_ = 2.832, *p* = 0.008). The positive regression coefficient here implies that a post-operative increase in decisional stochasticity was observed in patients who had a larger post-operative decrease in LEDD (Fig. 4). However, this relationship appeared to be driven by an outlier participant with a particularly large perioperative decrease in LEDD. When this participant was removed, the relationship was no longer statistically significant. We have removed the outlier in Fig. 4 but a full plot including the outlier can be found in Supplementary Fig. 5.

## Discussion

In this study, we employed a naturalistic gambling task and a hierarchical Bayesian model (for inference on subject-specific estimates of uncertainty) in order to investigate impulsive decision-making in participants with PD undertaking subthalamic DBS. Gambling behaviour associated with a ‘gold-standard’ questionnaire (BIS) measure of impulsivity, with post-DBS changes in gambling behaviours indexing postoperative changes in impulsivity. We also found that parameter estimates representing subjective estimates of environmental uncertainty (volatility) changed significantly from pre- to postoperative conditions. In particular, there was a significant increase in *ω*, that reflects a gambler’s estimate of how quickly the probability of winning on a given slot machine was changing (volatility), There was also a postoperative decrease in a second parameter, *β*, that captures the decision noise in a player’s belief-to-choice mapping. Notably, these model-based estimates of uncertainty related to postoperative impulsivity. The greater the postoperative estimate of *ω*, the greater the postoperative BIS score. In other words, the more a participant perceived the pay-out tendency of a slot machine to be changing after DBS, the more impulsive they rated themselves. Additionally, the higher the pre- to postoperative decrease in estimates of *β*, the higher the postoperative increase in BIS score across six months of longitudinal follow up. In other words, the more a participant became indiscriminate in their belief-to-choice mapping after DBS, the more impulsive they rated themselves.

Our gambling task utilised a multivariate response variable (bet increase, machine switch, casino switch and double-up) that captured different aspects of impulsivity and explorative behaviour. Furthermore, by employing a generative model that mapped observed responses to perceptual states, we were able to infer directly upon subject-specific parameters defining individual differences in uncertainty encoding. This is an important point of difference from a purely behavioural analysis, in which responses can have more than one (ambiguous) proximate cause. In the HGF, parameters are mathematically defined and have a concrete influence upon learning at different levels of the hierarchy (see Mathys *et al*. 2011, 2014 for simulations that demonstrate these effects)^34,35^.

What is the significance of individual differences in uncertainty encoding? Increased estimates of environmental uncertainty accelerate the rate of learning at higher hierarchical levels, which could engender maladaptive learning at lower levels of the hierarchy. A high learning rate suppresses the influence of top-down expectations, and may impair learning about probabilistically aberrant events. In a recent investigation employing the HGF to model surprise about unexpected events, persons with autism learned more quickly about environmental volatility than controls without autism^66^. However, at lower levels of the hierarchy, the tendency to believe that environmental instability is unstable resulted in smaller prediction errors (surprise) when events violated expectations. In other words, when the world is judged to be unstable and unpredictable, an agent differentiates less between expected and unexpected outcomes. This offers a similar but computationally distinct account of the stimulation-related learning changes described in a previous study^17^, in which reduced positive and negative instrumental outcome sensitivity was reported as a consequence of neurostimulation. Similar to prior work, we found a positive relationship between model-based estimates of uncertainty and impulsivity^19,20,22,23^. A plausible computational account of impulsivity is that high subjective uncertainty leads to lack of predictability and thus increases a tendency for short-term reward seeking and exploration.

We established that the ‘standard’ HGF best explained the gambling behaviour of our participants, in favour of a Rescorla-Wagner model or an ‘uncertainty-driven’ HGF. Importantly, the distinction between the ‘standard’ and ‘uncertainty-driven’ HGF models pertains only to the modelling of responses (the perceptual model is identical), in which the ‘standard’ HGF employs a fixed decision temperature and the ‘uncertainty-driven’ HGF a dynamic belief-to-response mapping based on online estimates of uncertainty (of beliefs about winning probability). These model comparison results suggest that our participants incorporate estimates of volatility into their prediction of reward probability but do not vary the stochasticity of their responses in response to these estimates. This is an interesting point of difference from the findings amongst younger, healthy males who completed a similar (albeit much longer) version of this task^19^ and future work will corroborate whether this finding of a static decision temperature is also observed amongst other neurodegenerative disorders.

In our participants, neurostimulation may interact with the physiology of the STN and alter the computations it implements. A tripartite functional organisation of the STN into limbic, associative and motor subregions is suggested by primate and human studies^67,68^, with electrode implantation targeted to the dorsolateral sensorimotor region to address motor symptoms of PD^69^. Yet, the small size of the STN means that dispersion of electrical charge from a stimulating contact in this region could still modulate subthalamic regions with greater connectivity to fronto-striatal networks. The more ventral and medial the stimulating contact, the more likely these networks are to be affected by DBS. Previous investigations have suggested that the site of subthalamic stimulation can modulate cognitive^70^ and psychiatric symptoms^49,71,72^. How could STN-DBS modulate uncertainty? From a computational perspective, the STN has been considered to implement a ‘delay’ on cognitive-associative circuits in the basal ganglia, allowing more information to be gathered to guide the most appropriate behavioural policy, suppressing impulsive and potentially error-prone responding^13,14^. It is possible that by modulating the decision threshold, STN-DBS could alter the bound for evidence accumulation and thus uncertainty in the representation of the reward environment^73,74^. Further work employing drift diffusion modelling to quantify rates of evidence accumulation and decision boundaries after STN-DBS may be illuminating, having previously helped to elucidate the mechanisms underlying hallucinations in PD^75^. Further work is also required to determine if the site of stimulation affects the magnitude of changes in uncertainty estimation observed here and specifically if cognitive-associative or sensorimotor regions of the STN are most implicated in these shifts.

We did not observe a cross-sectional relationship between dopaminergic medication (expressed as LEDD) and uncertainty encoding, nor did we observe a longitudinal relationship between LEDD and self-reported impulsivity. However, there was a longitudinal relationship between changes in model parameter estimates and the maximum reduction in LEDD during longitudinal follow up. Specifically, the greater the increase in decision noise (the greater the decrease in *β*), the greater the postoperative reduction in LEDD. It is difficult to be certain about whether this is a causal relationship and it may be an epiphenomenon of effective subthalamic DBS: One of the benefits of the STN (as opposed to other surgical targets in DBS for PD such as the internal segment of the globus pallidus) is that it allows for significant postoperative reduction in dopaminergic medication. Therefore, this apparent relationship could well be mediated by the effect of electrical stimulation – increasing indiscriminate responding and leading to a reduced requirement for dopaminergic therapy. Moreover, the finding that this relationship no longer held after the removal of an outlying participant decreases the confidence in this result.

There are likely to be fundamental differences in the computational operations subserved by the STN and dopamine in decision-making and impulsive behaviour. We have discussed the chronometric role of the STN is setting a decision bound and delaying impulsive choice, whereas dopamine is likely to have an essential role in reinforcement learning and reward evaluation^76–83^. In a non-surgical population, persons with PD withdrawn from medication display a characteristic impairment in reward learning and may show enhanced punishment sensitivity^84^. However, whilst dopamine replacement enhances the ability to learn from positive outcomes, learning from negative outcomes is impaired^84^. Thus, if postoperative LEDD reduction were a principal driver of a change in behaviour subsequent to DBS, then a selective impairment in positive outcome representation would be expected. However, from the HGF perspective, an agent with increased uncertainty at higher levels would be expected to show both decreased reward and punishment learning, as surprise to both positive and negative unexpected outcomes would be reduced (see Lawson *et al*.)^66^. This suggests that LEDD changes may have a secondary role, but further careful experiments will be necessary to address this question. For example, the goal of this behavioural analysis was to relate a computational marker of uncertainty (over all trials) to impulsivity, but future neuroimaging investigations could model trial-wise positive and negative reward prediction errors and relate this to trial-wise brain activity. Participants could also be tested prior to STN-DBS ‘on’ and ‘off’ medication (although in our cohort, participants were too impaired by their movement disorder to tolerate this and a group of less severely-affected individuals would be required).

We did not observe significant correlations between behaviour or parameters inferred from slot machine play with other estimates of impulsivity including the excluded letter fluency task, the Hayling test and the delay discounting task. This reflects the multifaceted nature of impulsivity, which may implicate discrete subcortical and cortical regions and may evidence differential patterns of expression amongst impulsive endophenotypes^85,86^. For example, the Hayling and ELF tasks are more commonly included amongst measures of task-switching and conflict interference, whilst the delay discounting task assesses impatience. Alternative paradigms may be required to capture participant-wise behaviour amongst these constructs.

We acknowledge methodological limitations of our investigation. The lack of a counterbalanced on-off stimulation design means that we cannot directly infer that stimulation underlies the observed changes in perceptual modelling observed in our participants (rather than, for example, practice effects or time). Specifically, for our participants the pre-DBS session was the first time they had performed the task, and so changes in postoperative behaviour could also be attributable to greater familiarity with the task and perhaps the inherent volatility of the reward structure. However, we suggest that a strength of our longitudinal design is that it is more reflective of the natural clinical course taken by persons with PD in the clinic. Moreover, our participants simply would not have tolerated an extended DBS washout and we hypothesise that the younger age of participants in the study of Seymour *et al*. may have facilitated their crossover design^17^. Nevertheless, it would be important to consider future experiments that could resolve this question, for example, selecting a cohort of younger PD participants who could tolerate a washout of stimulation, or testing a cohort of PD participants without DBS twice, 13-weeks apart.

Unfortunately, in this study, we were unable to utilise estimates of meta-volatility in our analysis as *ϑ* could not be robustly recovered from simulated data. This failure to recover *ϑ* might result from the limited number of trials (100) completed by each participant, which limits the amount of information that can be gathered to update estimates of this higher-level HGF parameter from the population prior. Again, the disability of our participant cohort prohibited a greater number of trials (as employed in previous studies using this paradigm)^19^, but this could be considered in future studies using younger or less severely-affected participants.

In summary, this study suggests that subjective estimates of uncertainty pertaining to environmental volatility and the stochasticity in belief-to-choice mapping change after subthalamic DBS for PD and relate significantly to postoperative impulsivity. Increased estimates of environmental uncertainty (volatility) and increased noise in the decision process may contribute to impulsivity as a clinically relevant form of maladaptive behaviour. Uncertainty elevates the learning rate and suppresses top-down expectations, which may blunt error signalling in a series of trial-wise outcomes. Similarly, a consistent decision rule with regards to acting on an internal model of the world is important to make appropriate decisions based on what has been learned. We therefore posit a cognitive mechanism for the genesis of impulsive behaviour in this population. Finally, our results demonstrate that a naturalistic assessment of gambling behaviour in a virtual casino is useful for investigating impulsivity in PD. The potential of our model to explain changes in impulsivity through game play could be most valuable in PD, given the significant, but poorly quantified risks relating to surgical (neurostimulation) and medical (dopamine agonist) treatments. If those at a higher risk of neuropsychiatric harm could be identified, this would improve the nature of treatment choice and informed consent and the effectiveness of clinical follow-up.

## Supplementary information

Supplementary Information

## Data Availability

The HGF toolbox is part of the open source TAPAS software and available for download at http://www.translationalneuromodeling.org/tapas. The gambling paradigm is provided for download on a git repository at https://github.com/saeepaliwal/breakspear_slot_machine.git. The analysis pipeline is provided at https://github.com/saeepaliwal/dbs_pd_analysis_pipeline.git. A de-identified data set containing neuropsychiatric assessment and gambling data can be provided by Dr Philip Mosley (Philip.Mosley@qimrberghofer.edu.au) on application, subject to institutional review board approval.

## References

[1] Vila M (2000). Evolution of changes in neuronal activity in the subthalamic nucleus of rats with unilateral lesion of the substantia nigra assessed by metabolic and electrophysiological measurements. The European journal of neuroscience.

[2] Brown P (2001). Dopamine dependency of oscillations between subthalamic nucleus and pallidum in Parkinson’s disease. The Journal of neuroscience: the official journal of the Society for Neuroscience.

[3] Schuepbach WM (2013). Neurostimulation for Parkinson’s disease with early motor complications. New England Journal of Medicine.

[4] Mosley PE, Marsh R (2015). The psychiatric and neuropsychiatric symptoms after subthalamic stimulation for Parkinson’s disease. Journal of Neuropsychiatry and Clinical Neurosciences.

[5] Jahanshahi M, Obeso I, Baunez C, Alegre M, Krack P (2015). Parkinson’s disease, the subthalamic nucleus, inhibition, and impulsivity. Movement disorders.

[6] Nambu A, Tokuno H, Takada M (2002). Functional significance of the cortico-subthalamo-pallidal ‘hyperdirect’ pathway. Neurosci Res.

[7] Aron AR, Behrens TE, Smith S, Frank MJ, Poldrack RA (2007). Triangulating a cognitive control network using diffusion-weighted magnetic resonance imaging (MRI) and functional MRI. The Journal of neuroscience: the official journal of the Society for Neuroscience.

[8] Rae CL, Hughes LE, Anderson MC, Rowe JB (2015). The prefrontal cortex achieves inhibitory control by facilitating subcortical motor pathway connectivity. The Journal of neuroscience: the official journal of the Society for Neuroscience.

[9] Hershey T (2004). Stimulation of STN impairs aspects of cognitive control in PD. Neurology.

[10] Obeso I, Wilkinson L, Rodriguez-Oroz MC, Obeso JA, Jahanshahi M (2013). Bilateral stimulation of the subthalamic nucleus has differential effects on reactive and proactive inhibition and conflict-induced slowing in Parkinson’s disease. Experimental Brain Research.

[11] Witt K (2004). Deep brain stimulation of the subthalamic nucleus improves cognitive flexibility but impairs response inhibition in Parkinson disease. Archives of Neurology.

[12] Thobois S (2007). STN stimulation alters pallidal-frontal coupling during response selection under competition. J Cereb Blood Flow Metab.

[13] Frank MJ, Samanta J, Moustafa AA, Sherman SJ (2007). Hold your horses: impulsivity, deep brain stimulation, and medication in parkinsonism. Science.

[14] Cavanagh JF (2011). Subthalamic nucleus stimulation reverses mediofrontal influence over decision threshold. Nature neuroscience.

[15] Florin E (2013). Subthalamic stimulation modulates self-estimation of patients with Parkinson’s disease and induces risk-seeking behaviour. Brain: a journal of neurology.

[16] Wagenbreth C (2015). Deep brain stimulation of the subthalamic nucleus modulates reward processing and action selection in Parkinson patients. J Neurol.

[17] Seymour B (2016). Deep brain stimulation of the subthalamic nucleus modulates sensitivity to decision outcome value in Parkinson’s disease. Sci Rep.

[18] Patton JH, Stanford MS, Barratt ES (1995). Factor structure of the Barratt impulsiveness scale. Journal of Clinical Psychology.

[19] Paliwal S, Petzschner FH, Schmitz AK, Tittgemeyer M, Stephan KE (2014). A model-based analysis of impulsivity using a slot-machine gambling paradigm. Frontiers in human neuroscience.

[20] FitzGerald TH, Schwartenbeck P, Moutoussis M, Dolan RJ, Friston K (2015). Active inference, evidence accumulation, and the urn task. Neural Comput.

[21] Averbeck BB, O’Sullivan SS, Djamshidian A (2014). Impulsive and compulsive behaviors in Parkinson’s disease. Annu Rev Clin Psychol.

[22] Djamshidian A (2012). Decision making, impulsivity, and addictions: do Parkinson’s disease patients jump to conclusions?. Movement disorders.

[23] Averbeck BB (2013). Uncertainty about mapping future actions into rewards may underlie performance on multiple measures of impulsivity in behavioral addiction: evidence from Parkinson’s disease. Behav Neurosci.

[24] Daunizeau J (2010). Observing the observer (I): meta-bayesian models of learning and decision-making. PloS one.

[25] Weiss Y, Simoncelli EP, Adelson EH (2002). Motion illusions as optimal percepts. Nature neuroscience.

[26] Petzschner FH, Glasauer S, Stephan KE (2015). A Bayesian perspective on magnitude estimation. Trends in cognitive sciences.

[27] Wolpert DM, Ghahramani Z, Jordan MI (1995). An internal model for sensorimotor integration. Science.

[28] Kording KP, Wolpert DM (2004). Bayesian integration in sensorimotor learning. Nature.

[29] Tenenbaum JB, Griffiths TL, Kemp C (2006). Theory-based Bayesian models of inductive learning and reasoning. Trends in cognitive sciences.

[30] Friston K (2009). The free-energy principle: a rough guide to the brain?. Trends in cognitive sciences.

[31] Griffiths TL, Chater N, Kemp C, Perfors A, Tenenbaum JB (2010). Probabilistic models of cognition: exploring representations and inductive biases. Trends in cognitive sciences.

[32] Tenenbaum JB, Kemp C, Griffiths TL, Goodman ND (2011). How to grow a mind: statistics, structure, and abstraction. Science.

[33] Behrens TE, Woolrich MW, Walton ME, Rushworth MF (2007). Learning the value of information in an uncertain world. Nature neuroscience.

[34] Mathys CD (2014). Uncertainty in perception and the Hierarchical Gaussian Filter. Frontiers in human neuroscience.

[35] Mathys C, Daunizeau J, Friston KJ, Stephan KE (2011). A bayesian foundation for individual learning under uncertainty. Frontiers in human neuroscience.

[36] Vossel S (2014). Spatial attention, precision, and Bayesian inference: a study of saccadic response speed. Cerebral cortex.

[37] Schwartenbeck P (2015). Optimal inference with suboptimal models: addiction and active Bayesian inference. Medical hypotheses.

[38] Lhommee E (2012). Subthalamic stimulation in Parkinson’s disease: restoring the balance of motivated behaviours. Brain: a journal of neurology.

[39] Mosley, P. E., Marsh, R., Perry, A., Coyne, T. & Silburn, P. Persistence of Mania After Cessation of Stimulation Following Subthalamic Deep Brain Stimulation. *Journal of Neuropsychiatry and Clinical Neurosciences* Published Online in Advance of Print (2018). 10.1176/appi.neuropsych.17060129 29458279

[40] Smeding HM (2007). Pathological gambling after bilateral subthalamic nucleus stimulation in Parkinson disease. Journal of Neurology, Neurosurgery, and Psychiatry.

[41] Lim SY (2009). Dopamine dysregulation syndrome, impulse control disorders and punding after deep brain stimulation surgery for Parkinson’s disease. Journal of clinical neuroscience: official journal of the Neurosurgical Society of Australasia.

[42] Halbig TD (2009). Subthalamic deep brain stimulation and impulse control in Parkinson’s disease. European journal of neurology: the official journal of the European Federation of Neurological Societies.

[43] Amami P (2015). Impulse control behaviours in patients with Parkinson’s disease after subthalamic deep brain stimulation: de novo cases and 3-year follow-up. Journal of Neurology, Neurosurgery, and Psychiatry.

[44] Voon V, Kubu C, Krack P, Houeto JL, Troster AI (2006). Deep brain stimulation: neuropsychological and neuropsychiatric issues. Movement disorders.

[45] Hughes AJ, Daniel SE, Kilford L, Lees AJ (1992). Accuracy of clinical diagnosis of idiopathic Parkinson’s disease: a clinico-pathological study of 100 cases. Journal of Neurology, Neurosurgery, and Psychiatry.

[46] Emre M (2007). Clinical diagnostic criteria for dementia associated with Parkinson’s disease. Movement disorders.

[47] Hoehn MM, Yahr MD (1967). Parkinsonism: onset, progression and mortality. Neurology.

[48] Mosley PE, Breakspear M, Coyne T, Silburn P, Smith D (2018). Caregiver burden and caregiver appraisal of psychiatric symptoms are not modulated by subthalamic deep brain stimulation for Parkinson’s disease. NPJ Parkinsons Disease.

[49] Mosley PE (2018). The site of stimulation moderates neuropsychiatric symptoms after subthalamic deep brain stimulation for Parkinson’s disease. NeuroImage: Clinical.

[50] Weintraub D (2012). Questionnaire for impulsive-compulsive disorders in Parkinson’s Disease–Rating Scale. Movement disorders.

[51] Kirby KN, Petry NM, Bickel WK (1999). Heroin addicts have higher discount rates for delayed rewards than non-drug-using controls. Journal of experimental psychology. General.

[52] Shores EA, Carstairs JR, Crawford JR (2006). Excluded Letter Fluency Test (ELF): Norms and Test–Retest Reliability Data for Healthy Young Adults. Brain Impairment.

[53] Burgess, P. W., Shallice, T. & Thames Valley Test Company. *The Hayling and Brixton tests*. (Thames Valley Test Company, 1997).

[54] Beck AT, Ward CH, Mendelson M, Mock J, Erbaugh J (1961). An Inventory for Measuring Depression. Archives of General Psychiatry.

[55] Baron-Cohen S, Wheelwright S (2004). The empathy quotient: an investigation of adults with Asperger syndrome or high functioning autism, and normal sex differences. Journal of Autism and Developmental Disorders.

[56] Pachana NA (2007). Development and validation of the Geriatric Anxiety Inventory. International Psychogeriatrics.

[57] Starkstein SE (1992). Reliability, validity, and clinical correlates of apathy in Parkinson’s disease. The Journal of neuropsychiatry and clinical neurosciences.

[58] Goetz CG (2007). Movement Disorder Society-sponsored revision of the Unified Parkinson’s Disease Rating Scale (MDS-UPDRS): Process, format, and clinimetric testing plan. Movement disorders.

[59] Evans AH (2004). Punding in Parkinson’s disease: its relation to the dopamine dysregulation syndrome. Movement disorders.

[60] Rescorla, R. A. & Wagner, A. W. In *Classical Conditioning II: Current* Research *and Theory* (eds Black, A. H. & Prokasy, W. F.) 64–99 (Appleton-Century-Crofts, 1972).

[61] Aponte EA (2016). mpdcm: A toolbox for massively parallel dynamic causal modeling. Journal of neuroscience methods.

[62] Brodersen KH (2014). Dissecting psychiatric spectrum disorders by generative embedding. Neuroimage Clin.

[63] Brodersen KH (2011). Generative embedding for model-based classification of fMRI data. PLoS Comput Biol.

[64] Stanford MS (2009). Fifty years of the Barratt Impulsiveness Scale: An update and review. Personality and Individual Differences.

[65] Voon V (2007). Factors associated with dopaminergic drug-related pathological gambling in Parkinson disease. Archives of Neurology.

[66] Lawson RP, Mathys C, Rees G (2017). Adults with autism overestimate the volatility of the sensory environment. Nature neuroscience.

[67] Haynes WI, Haber SN (2013). The organization of prefrontal-subthalamic inputs in primates provides an anatomical substrate for both functional specificity and integration: implications for Basal Ganglia models and deep brain stimulation. The Journal of neuroscience: the official journal of the Society for Neuroscience.

[68] Lambert C (2012). Confirmation of functional zones within the human subthalamic nucleus: patterns of connectivity and sub-parcellation using diffusion weighted imaging. NeuroImage.

[69] Wodarg F (2012). Stimulation site within the MRI-defined STN predicts postoperative motor outcome. Movement disorders.

[70] Hershey T (2010). Mapping Go-No-Go performance within the subthalamic nucleus region. Brain: a journal of neurology.

[71] Mallet L (2007). Stimulation of subterritories of the subthalamic nucleus reveals its role in the integration of the emotional and motor aspects of behavior. Proceedings of the National Academy of Sciences of the United States of America.

[72] Welter ML (2014). Optimal target localization for subthalamic stimulation in patients with Parkinson disease. Neurology.

[73] Pote I (2016). Subthalamic nucleus deep brain stimulation induces impulsive action when patients with Parkinson’s disease act under speed pressure. Experimental brain research.

[74] Herz DM (2018). Mechanisms Underlying Decision-Making as Revealed by Deep-Brain Stimulation in Patients with Parkinson’s Disease. Current biology: CB.

[75] O’Callaghan C (2017). Visual Hallucinations Are Characterized by Impaired Sensory Evidence Accumulation: Insights From Hierarchical Drift Diffusion Modeling in Parkinson’s Disease. Biol Psychiatry Cogn Neurosci Neuroimaging.

[76] Schultz W, Dayan P, Montague PR (1997). A neural substrate of prediction and reward. Science.

[77] Kishida KT (2016). Subsecond dopamine fluctuations in human striatum encode superposed error signals about actual and counterfactual reward. Proceedings of the National Academy of Sciences of the United States of America.

[78] Haber SN, Knutson B (2010). The reward circuit: linking primate anatomy and human imaging. Neuropsychopharmacology.

[79] Basar K (2010). Nucleus accumbens and impulsivity. Prog Neurobiol.

[80] Wittmann BC, Daw ND, Seymour B, Dolan RJ (2008). Striatal activity underlies novelty-based choice in humans. Neuron.

[81] Tanaka SC, Balleine BW, O’Doherty JP (2008). Calculating consequences: brain systems that encode the causal effects of actions. The Journal of neuroscience: the official journal of the Society for Neuroscience.

[82] Abler B, Walter H, Erk S, Kammerer H, Spitzer M (2006). Prediction error as a linear function of reward probability is coded in human nucleus accumbens. NeuroImage.

[83] Daw ND, O’Doherty JP, Dayan P, Seymour B, Dolan RJ (2006). Cortical substrates for exploratory decisions in humans. Nature.

[84] Frank MJ, Seeberger LC, O’Reilly RC (2004). By carrot or by stick: cognitive reinforcement learning in parkinsonism. Science.

[85] Nombela C, Rittman T, Robbins TW, Rowe JB (2014). Multiple modes of impulsivity in Parkinson’s disease. PloS one.

[86] Robbins TW, Gillan CM, Smith DG, de Wit S, Ersche KD (2012). Neurocognitive endophenotypes of impulsivity and compulsivity: towards dimensional psychiatry. Trends in cognitive sciences.

