# Supplementary material for: Subjective estimates of uncertainty during gambling and impulsivity after subthalamic deep brain stimulation for Parkinson’s disease
Source: Sci Rep. 2019 Oct 15;9:14795. doi: 10.1038/s41598-019-51164-2 (PMC6794275; doi:10.1038/s41598-019-51164-2)
Supplement: Supplementary file 1 — Supplementary Information [file 41598_2019_51164_MOESM1_ESM.pdf]

Supplementary Material

Subjective estimates of uncertainty during gambling and impulsivity after subthalamic deep brain stimulation for Parkinson's disease

Saee Paliwal [1] *, Philip E Mosley [2,3,4,5] *, Michael Breakspear [2], Terry Coyne [4,6], Peter Silburn [3,4], Eduardo Aponte [1], Christoph Mathys [1,9], Klaas E. Stephan [1,7,8]

[1] *Translational Neuromodeling Unit (TNU), Institute for Biomedical Engineering, University of Zürich and Swiss Federal Institute of Technology (ETH Zürich), Zürich, Switzerland*
[2] *Systems Neuroscience Group, QIMR Berghofer Medical Research Institute, Herston, Queensland, Australia*
[3] *Neurosciences Queensland, St Andrew's War Memorial Hospital, Spring Hill, Queensland, Australia*
[4] *Queensland Brain Institute, University of Queensland, St Lucia, Queensland, Australia*
[5] *Faculty of Medicine, University of Queensland, Herston, Queensland, Australia*
[6] *Brizbrain and Spine, the Wesley Hospital, Auchenflower, Queensland, Australia*
[7] *Max Planck Institute for Metabolism Research, Cologne, Germany.*
[8] *Wellcome Trust Centre for Neuroimaging, University College London, London, UK*
[9] *Scuola Internazionale Superiore di Studi Avanzati (SISSA), Trieste, Italy*
* These authors contributed equally to the work

* These authors contributed equally to the work

Correspondence to:
Dr Philip E Mosley, Neurosciences Queensland, Level 1, St Andrew's Place, 33 North Street, Spring Hill, Queensland, 4000, Australia

E-mail: philip.mosley@qimrberghofer.edu.au

Telephone: +61 (7) 3839 3688

1. Supplementary Material

1.1 Investigation Timeline

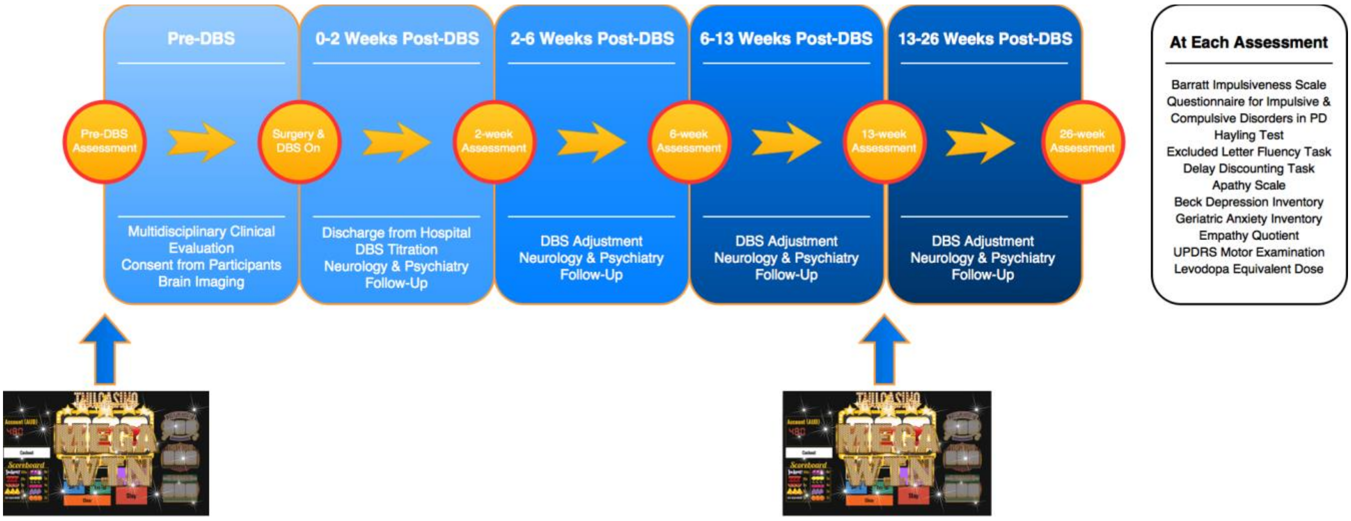

Supplementary Figure 1 | Overview of Study Timeline: Persons with PD were screened in a multidisciplinary clinic by a movement disorders neurologist, neurosurgeon, psychiatrist, and rehabilitation specialist prior to selection for subthalamic DBS. Prior to surgery, surgical candidates who consented to participate were assessed with a battery of neuropsychiatric instruments (see box). These instruments were repeated at 2-weeks, 6-weeks, 13-weeks and 26-weeks postoperatively. This methodology of iterative assessments was designed to capture fluctuations in neuropsychiatric symptoms as electrical stimulation was titrated and dopaminergic medication was reduced. The slot machine gambling paradigm was undertaken by participants prior to neurosurgery and at 13-weeks postoperatively.

1.2 Neuropsychiatric Assessment Instruments

1.2.1 Barratt Impulsiveness Scale

The BIS is one of the most widely used instruments for assessing trait impulsivity and is often the 'gold standard' instrument against which other measures are compared. It is a 30-item self-report questionnaire assessing the prevalence of impulsive behaviours. Respondents must rate each item (e.g. '*I act on the spur of the moment*') from 1 to 4 according to the frequency of occurrence (i.e. rarely / never; occasionally; often; always / almost always). Higher scores indicate greater impulsivity. The mean BIS score is significantly greater in PD patients with ICDs compared to non-impulsive PD patients,[1,2] suggesting that this instrument also has construct validity in the assessment of impulsivity in PD.

1.2.2 Questionnaire for Impulsive-Compulsive disorders in PD

The QUIP-RS is a 28-item self-report questionnaire assessing the prevalence of ICDs including compulsive spending, hypersexuality, pathological gambling, binge eating, hobbyism, punding and dopamine dysregulation. Respondents much rate each item (e.g. '*Do you have urges or desires for the following behaviours that you feel are excessive or cause you distress?*') from 0 (never) to 4 (very often). The total sum obtained for each compulsive behaviour indicates the current severity of that behaviour. The instrument was designed for use in the PD population and was previously validated against a semi-structured clinical interview.

1.2.3 Hayling Test

The Hayling Test is a sentence completion task, during which participants must insert a nonsense word at the end of a sentence, inhibiting the pre-potent stimulus to complete the sentence with a word that makes sense. The test assesses the construct of inhibition and is sensitive to frontal lobe dysfunction. For example, in the sentence: '*the whole town came to hear the Mayor…*' a correct response could be '*banana*'. Participants would be penalised for completing the sentence with the clearly related words '*speak*', or '*talk*' (referred to as category A errors), as well as with words that are only partially connected such as '*explode*' (referred to as category B errors). PD patients make more category A and B errors than controls on this task.[3,4]

1.2.4 Excluded Letter Fluency Task

The ELF is an additional measure of inhibitory control. Participants are given 3 trials of 90 seconds to produce as many words as possible that do not contain a specified vowel. Words must be longer than 3 letters and cannot be proper nouns or derivations of the same word stem. Scoring includes an overall correct total, the number of rule violations and the number of word repetitions. In a sample of 50 PD patients, the number of rule violations was previously shown to be significantly greater compared to age-matched controls and was highly correlated with anatomical changes in brain regions implicated in inhibition.[5]

1.2.5 Delay Discounting Task

An assessment of delay aversion, the tendency to prefer sooner, smaller rewards over those that are larger but temporally more distant. The task was designed to assess impulsivity in individuals with substance use disorders; behaviours that share face validity with the impulse-control disorders (ICDs) observed in a subset of PD patients. Participants are presented with a series of 27 choices between an

amount of money distributed immediately and a larger sum after a specified delay. After the task is complete, participants have the opportunity to win the amount of money they have chosen in a choice selected at random, either immediately or after a delay, depending on the choice they have made. Subsequently, the pattern of choices is analysed to calculate a discount parameter, or the point of indifference between delayed and immediate rewards for a given sum. Individuals with greater delay aversion have a higher discount parameter. The extent of delay aversion was previously shown to be greater amongst PD patients than healthy controls,[6] as well as being greater amongst PD patients with ICDs than non-impulsive PD patients.[7,8]

1.3 Slot Machine Gambling Paradigm: Game Trace

The slot-machine gambling paradigm employed in this study is a variant of the paradigm developed in Paliwal, Petzschner *et al*.[9] The task is designed to have standard features normally attributed to slot machines (colours, sounds, banners), and its features were designed to mirror the specifications of Swiss and German slot machines. Due to its game-like feel, the task successfully elicits impulsive, risk-taking and exploratory behaviour in participants. Task behaviour has been previously shown to correlate with standard measures of impulsivity (i.e. the BIS). Players begin the slot machine with 2000 AUD in their account, and play through 100 trials. The trajectory of win-loss outcomes is predetermined, ensuring that participants' experience of rewards and losses were comparable in order and quantity. The trajectory results in a positive outcome (net winnings) for most participants. At the end of the task, participants are awarded up to 30 AUD in real money based on the size of these virtual winnings.

The task begins with 5 training trials, after which the subject plays through the main task, consisting of 100 trials. Only data from the main task are used for further analysis. For the main task, the win probability is 25%, with wins split into big wins (12% of trials) and small wins (88% of trials). Players win when all three wheels show the same symbols (e.g. all three wheels display an apple image). There are two possible types of losses. The first is a near-miss, in which the first two wheels of the slot machine display the same symbol, and the third is different (e.g. cherry, cherry, apple). The second is a true loss, in which all the wheels display different images (e.g. cherry, apple, orange).

Game play proceeds as follows: at the onset of each trial, the main screen loads, displaying the player's account value. Players are then able to execute one of the following actions: place a bet (of unlimited magnitude – by loading the machine in increments of 5-10 AUD), switch slot machines, or 'cash out', which involves 'exiting' the casino and returning again on another virtual 'day'. If the player chooses to bet, after loading the machine, they press the 'Pull' button and watch as the wheels begin to spin. The player has the option of pressing the 'Stop' button at any time during wheel spin, ending the trial

and subsequently revealing the outcome of the three wheels, Pressing the stop button has no effect on the trial outcome; though this is not told to the participant. In the absence of the stop button being pressed, the trial times out after 5 seconds, and reveals the outcome to the player, with the first, second and third wheel stopping sequentially. For winning trials, there are ten possible reward amounts. Each possible reward is called a *reward grade*, and indicates a different multiple of the bet size placed (e.g. reward grade 1 indicates a reward amount that is double the bet amount placed). After each win trial, the player is offered a 'double-up' option, during which they are given 3 seconds to decide whether or not to engage in a 'double-or-nothing' option. The double-or-nothing option has two possible outcomes: if the player wins the double-or-nothing gamble, they double their win amount from that trial, if they lose the double-or-nothing option, they lose their entire win amount from the corresponding trial. If the player does nothing, or decides not to gamble, they are taken to the next trial. For losses, players are taken directly to the beginning of the next trial.

In the context of the analyses presented, this version of the slot machine deviates from the version presented in Paliwal, Petzschner *et al* in several important ways: here, players are given the ability to place unlimited bet sizes with the ability to increase their bets in increments of 5 or 10 AUD; there are no 'fake win' results, simply wins and losses; the task is considerably (50%) shorter; and finally, the task was aesthetically remodelled in order to be more naturalistic.

Each trial in the game follows a pre-programmed result sequence consisting of the following trial types:

- big wins: top three out of 10 reward grades
- small wins: lower 7 out of 10 reward grades
- near misses: when the same symbol appears in wheel 1 and two, and a different symbol appears in wheel 3
- true losses (all three wheels show different symbols).

After all win trials, a player is allowed to engage in a secondary double-or-nothing gamble, and has three seconds to decide whether or not to do so. A more detailed trial breakdown across the various types of wins and losses is listed below:

- 25 out of 100 trials are wins
- 3 out of 100 trials are big wins
- 22 out of 100 trials are small wins
- 22 out of 100 trials are near misses
- 52 out of 100 trials are full losses.

- All win trials contain a double-up option

1.4 Slot Machine Gambling Paradigm: Perceptual and Response Variables

The perceptual variable used in our modelling approach is a representation of win or loss in the task. Although the task itself allows for continuous valued rewards, for this analysis, we look at wins and losses as binary outcomes (big wins and small wins are collapsed for the purpose of model fitting). We do so for the following reasons: first, we do not measure reward sensitivity on an individual basis, therefore creating a parametric multimodal reward variable that is consistent across the entire population is difficult. Secondly, measuring uncertainty-updating and impulsive responses in reaction to a binary win/loss event allows for a clearer interpretation of the model parameters and the correlational results. Finally, the analysis presented in this paper is an application of the methodology presented in Paliwal, Petzschner *et al.*, and for this reason, we wanted to adhere as closely as possible to the modelling decisions made in this prior work.

The response variable used in the model is constructed from four key behaviours afforded to participants as they play the slot machine: bet behaviour, machine switching, doubling up and cashing out. Similar to the analyses done in Paliwal, Petzschner *et al*, we identify four key indicators of risk-taking within these behaviours:

- Bet Increase (BI)
  - 1 = switching from a low to a high bet
  - 0 = staying at the same bet size
- Double Up (DU)
  - 1 = engaging in the secondary double-or-nothing option
  - 0 = declining to engage in the secondary double-or-nothing option
- Casino Switch (CS)
  - 1 = switching casino days
  - 0 = remaining in the current casino day
- Machine switch (MS)
  - 1 = deciding to switch to a new machine
  - 0 = continuing to play on the same machine

While these actions might at first glance appear to relate to different behaviours, they all share a common theme in that they enhance outcome variance and thus risk (compare the definition of 'risk' in behavioural economics). For example, for a machine switch, regardless of whether the player is

performing well or poorly on the current machine, the decision to switch machines incurs the risk that the new machine chosen may be punishing or rewarding, thereby making the player vulnerable to the variance of the task. Similarly, a bet increase is a risk-inducing shift in the face of uncertainty, again making the player more susceptible to larger wins and losses. And in the same respect, casino switches and double-ups again expose players to the risk that their environment will change dramatically, and for the worst. Each of the above actions thus leads to greater outcome variance (risk), and risk-taking, in turn, is one critical component of impulsivity.[10] In order to combine these behaviours into a single representation of risk taking, we construct a trial-wise, binary response variable per participant by performing an OR operation over the four actions. If a subject performs one or more of these actions on a given trial, the response variable on that trial is a 1. If the subject did not engage in any of these actions on a given trial, the response variable on that trial is a 0.

Supplementary Table 1 | Composition of perceptual and response models for modelling

Composition of model variables	Win	Near-miss	True loss	BI	DU	CS	MS
**Perceptual variables**							
	**1**	**0**	**0**	**-**	**-**	**-**	**-**
**Response Variables**							
	**-**	**-**	**-**	**1**	**1**	**1**	**1**

Variables are binary and composed on a trial-by-trial basis for each of combinations shown. BI, Bet increase; DU, double-up; CS, casino switch; MS, machine switch. Wins are encoded as 1, losses as 0.

1.5 Formal Summary of the Hierarchical Gaussian Filter

The goal of the HGF is to infer how an individual subject learns about hierarchically coupled environmental quantities under different forms of uncertainty (including volatility). In our case, the first quantity, x_1, represents trial-wise outcomes (wins or losses) in the slot machine. This derives, through a sigmoid transform, from a second-level variable, x_2 which represents, in logit space, the probability of winning (an indication of the slot-machine being 'hot' or 'cold'). The variable x_2 performs a Gaussian random walk trial by trial, with its step size (or variance) coupled to a higher level x_3 (the speed at which a machine fluctuates between 'hot' and 'cold' states), according to $f_2(x_3)$. The coupling between levels follows an expansion of $\log f(x)$ to first order, with subject-specific parameters κ, ω that determine the individual learning and inference style (see Equation 5 below). Generated observations, u, deterministically depend on x_1, i.e., there is no sensory noise. A detailed derivation of the exact equations can be found in Mathys *et al* (2014).[11] Here, we provide a very brief summary only.

For model inversion and estimating posterior distributions of states and parameters, the HGF employs a generic variational Bayesian approximation, assuming that the posterior distributions of the states are Gaussian at all levels of the hierarchy:

$$x_i^{(k)} | u^{(1)}, \ldots, u^{(k)}, \chi \sim \mathcal{N}\left(\mu_i^{(k)}, \left(\pi_i^{(k)}\right)^{-1}\right), \qquad (1)$$

where u is an observed input, $\mu_i^{(k)}$ is the mean at time point k for level i, and $\pi_i^{(k)}$ is the precision, or inverse variance, of this distribution, and $\chi \stackrel{\text{def}}{=} \{\kappa, \omega, \vartheta\}$ are subject-specific parameters. Updates to the posterior mean $\mu_i^{(k)}$ for level i have the general form:

$$\Delta\mu_i \propto \frac{\hat{\pi}_{i-1}}{\pi_i} \delta_{i-1} \qquad (2)$$

Thus, updates to the posterior mean at each level of the HGF are proportional to the prediction error (PE) at the level below, δ_{i-1}, weighted by a ratio of uncertainties (or their inverses, precisions). Specifically, this ratio consists of the precision of the prediction onto the level below, $\hat{\pi}_{i-1}$, and the posterior precision at the current level, $\pi_i^{(k)}$.This equation reveals that the precision ratio corresponds to a dynamic learning rate: the higher the precision (the lower the uncertainty) of a prediction on the level below, the more meaningful the input from the level below and the greater the impact of the PE on updating the posterior mean. Conversely, the more certain an agent is about the true value of x_i, the smaller the impact of the PE.

At the bottom of the hierarchy, the prediction error $\delta_u^{(k)}$ (= $\delta_0^{(k)}$) represents a value PE, the difference between the actual input u and the predicted input:

$$\delta_0^{(k)} = \delta_u^{(k)} \stackrel{\text{def}}{=} u^{(k)} - s\left(\mu_1^{(k-1)}\right) \qquad (3)$$

where s denotes the sigmoidal transform. At higher levels of the HGF, the PEs refer to volatility rather than value. A volatility PE (VOPE) integrates both predicted and observed, as well as informational and environmental uncertainty,

$$\delta_i^{(k)} = \frac{\sigma_i^k + (\mu_i^{(k)} - \mu_i^{(k-1)})^2}{\sigma_i^{(k-1)} + v_i^{(k)}} - 1, \qquad (4)$$

where $v_i^{(k)}$ is defined as,

$$v_i^{(k)} \stackrel{\text{def}}{=} exp(\kappa\mu_i^{(k-1)} + \omega) \qquad (5)$$

Here, informational uncertainty is given by $\sigma_i^{(k-1)}$, the variance of the posterior at trial k-1. Predicted total uncertainty is given by the denominator and includes both informational and environmental components. The environmental uncertainty represented by $v_i^{(k)}$ can be separated into phasic $\left(\kappa\mu_i^{(k-1)}\right)$ and tonic (ω) components, corresponding to 'unexpected' uncertainty related to environmental fluctuations. Additionally, v also affects the precision (or uncertainty) ratio (Equation 2) that defines the learning rate (for details, see Mathys et al. 2014). Because the HGF can react sensitively to sudden shifts in environmental contingencies by adjusting the phasic component of its volatility estimate, it allows learning rates to be adjusted to the time-varying statistical structure of the environment. Individual differences in this dynamic learning process are determined by the subject-specific uncertainty parameters κ, ω and ϑ.

1.6 Model Inversion

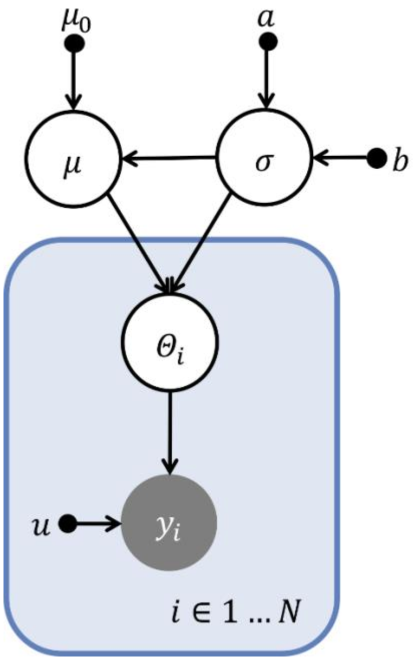

Supplementary Figure 2 | Schematic of hierarchical model inversion using population Markov-chain Monte Carlo sampling. Here, u represents the fixed perceptual variable that a participant observes during game play, and y_i represents the response variable for participant *i*. $\Theta_i = \{\kappa, \omega, \vartheta, \beta\}$ is a participant-specific parameter vector consisting of the perceptual parameters shown in Figure 2 and the decision temperature, β. μ and σ are the mean and variance of the group-level empirical prior that is constructed using observations from all *N* participants. This prior is simultaneously used to invert models on a participant-specific level, for example, using u and y_1, as the perceptual and response variables for Participant 1. In the notation used in this schematic, points indicate fixed parameters such as b or fixed data vectors such as u. Filled circles represent observed quantities, such as y_i. Unfilled circles represent random variables that are estimated during model inversion. The hyperparameter for μ, μ_0, is fixed to -3 for $\log\omega$ and -6 for $\log\vartheta$, before inversion. The hyperprior on σ is a gamma distribution with parameters a and b, which are also fixed before inversion. As in previous work (see Paliwal, Petzschner et al, 2014),[9] we did not estimate κ due to conditional dependencies with other parameters, but set it to unity.

1.7 The Rescorla-Wagner Model

The Rescorla-Wagner model is a simple associative learning model with the following form:

$$V^{(k)} = V^{(k-1)} + \alpha\left(\lambda^{(k)} - V^{(k-1)}\right) \quad (6)$$

where $V^{(k)}$is the state of the tracked variable, in our case, the win probability, at time k, $V^{(k-1)}$ is the state of the variable at time point *k-1*, α is the learning rate and $\lambda^{(k)}$ is the actual outcome at time point *k.* We combine this perceptual model with the same sigmoidal response model as used for the HGF.

1.8 Neuropsychiatric Assessment Data Pre- and Post-DBS

Supplementary Table 2: Additional Neuropsychiatric Assessment Data Pre- and Post-DBS

	**Pre-DBS**	**Post-DBS**	**Max Impairment**	**Pre- vs. Post-DBS**	
**Behavioural Measure**	*Mean (SD), Median (Range)*			*t-stat*	*p-value*
BIS Attentional	16.1 (±3.2),<br>16 (10 - 23)	15.0 (±3.4),<br>15 (8 - 22)	0.39 (±2.8),<br>0 (-5 - 5)	2.64	0.06
BIS Non-Planning	23.0 (±4.1),<br>23 (14 - 33)	22.4 (±5.2),<br>22 (15 - 36)	2.3 (±3.7),<br>2 (-6 - 9)	1.09	0.84
BIS Motor	21.2 (±3.3),<br>21 (14 - 29)	20.3 (±3.4)<br>21 (13 - 30)	1.2 (±3.5),<br>1 (-7 - 14)	1.87	0.28
Apathy Scale	11.5 (±5.7)<br>11 (1 - 26)	11.3 (±5.5)<br>11 (1 - 23)	2.8 (±4.2),<br>2 (-3 - 14)	0.30	1.54
Empathy Quotient	40.7 (±13.1)<br>39.5 (16 – 68)	40.4 (± 14.3)<br>36 (14 – 68)	-4.7 (±7.9),<br>-5 (-26 - 13)	0.27	1.54
Geriatric Anxiety Inventory	4.9 (±4.3),<br>5 (0 - 17)	3.0 (±4.0),<br>2 (0 - 20)	0.55 (±3.4),<br>0 (-5 - 11)	3.62	<0.001*

***p<0.001, **p<0.01, *p<0.05 *where p-values are Holm-Bonferroni corrected for multiple comparisons with* $\alpha = 0.05$.

1.9 Heterogeneity in Participant-Wise Trajectories

The participant-wise trajectories of key variables were inspected across all intervals in the investigation. Representative figures are presented below. Although mean changes were not significantly different at postoperative assessments, there was observable inter-individual heterogeneity in postoperative course.

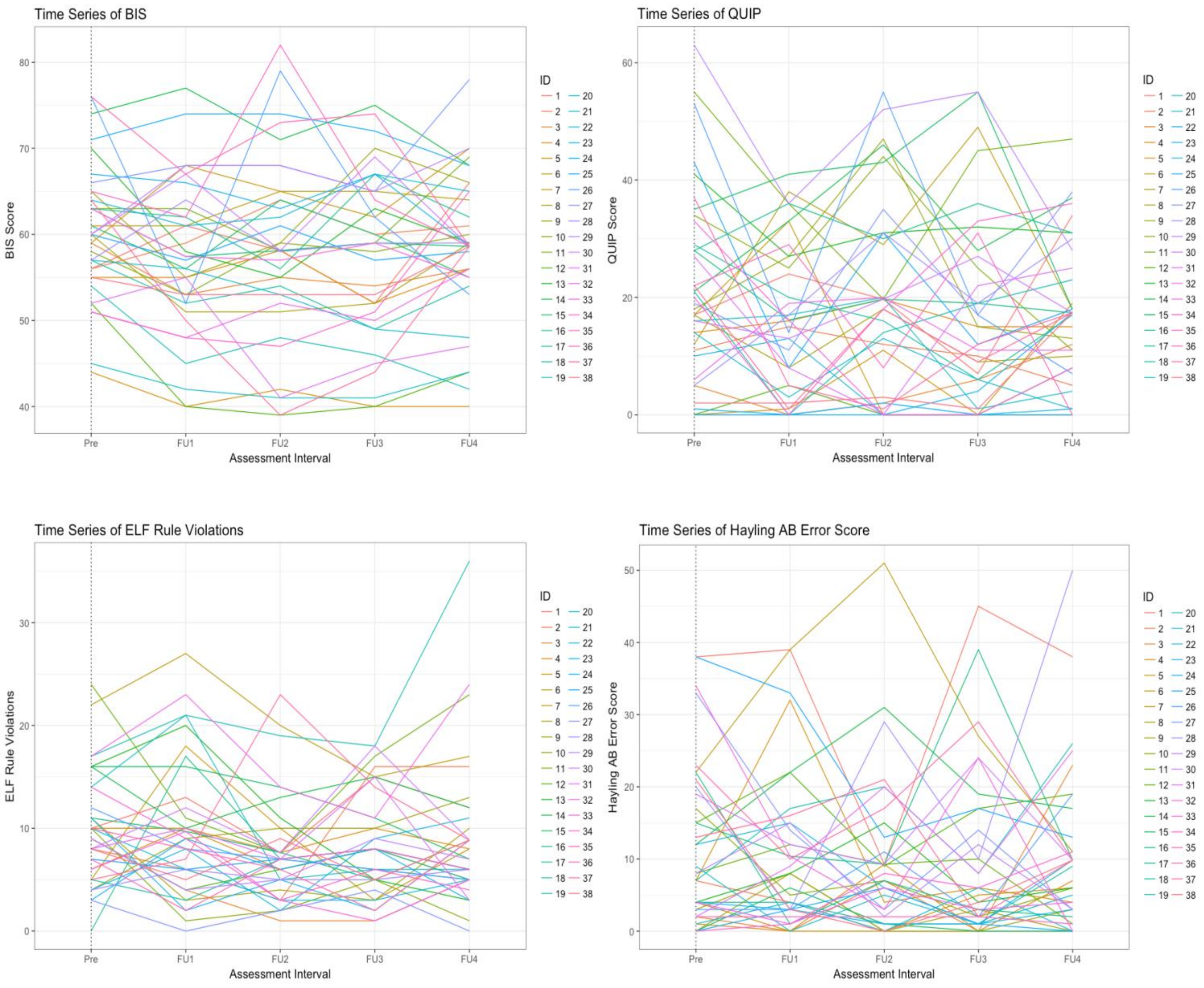

Supplementary Figure 3 | Heterogeneity in Participant-Wise Trajectories: BIS = Barratt Impulsiveness Scale, QUIP = Questionnaire for Impulsive-Compulsive Disorders in PD, ELF = Excluded Letter Fluency. Pre = Pre-DBS, FU1 = 2-weeks post-DBS, FU2 = 6-weeks post-DBS, FU3 = 13-weeks post-DBS, FU4 = 26-weeks post-DBS.

1.10 Parameter Recoverability

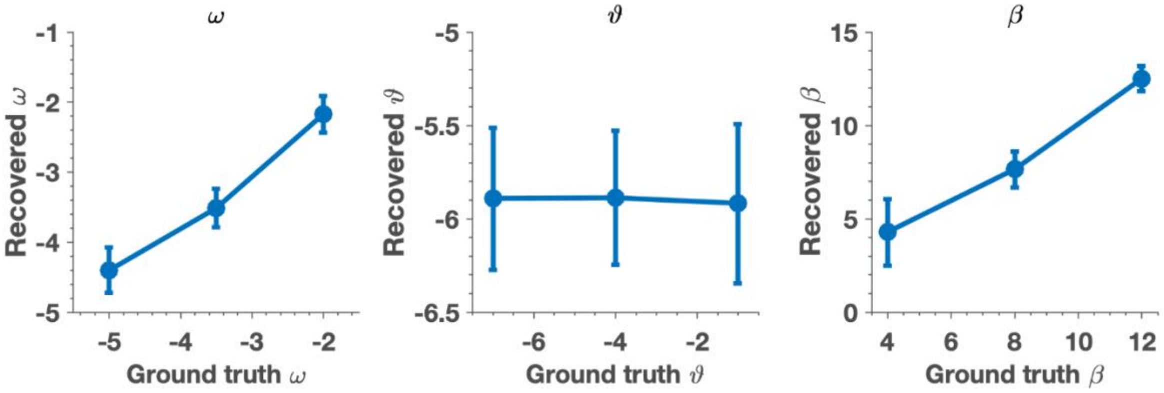

Supplementary Figure 4 | Parameter recoverability in the HGF. The first plot above shows the mean of the recovered parameter values for ω across 10 inversions of the HGF on the y-axis, against the ground truth ω values on the x-axis. The second plot shows the mean of recovered parameter values for ϑ across 10 inversions of the HGF on the y-axis, against the ground truth ϑ values on the x-axis. The third plot shows the mean of the recovered parameter values for β across 10 inversions of the HGF on the y-axis, against the ground truth parameters for β on the x-axis. Error bars indicate the standard deviation of the recovered parameter estimates across 10 inversions.

1.11 Computational Model Parameters

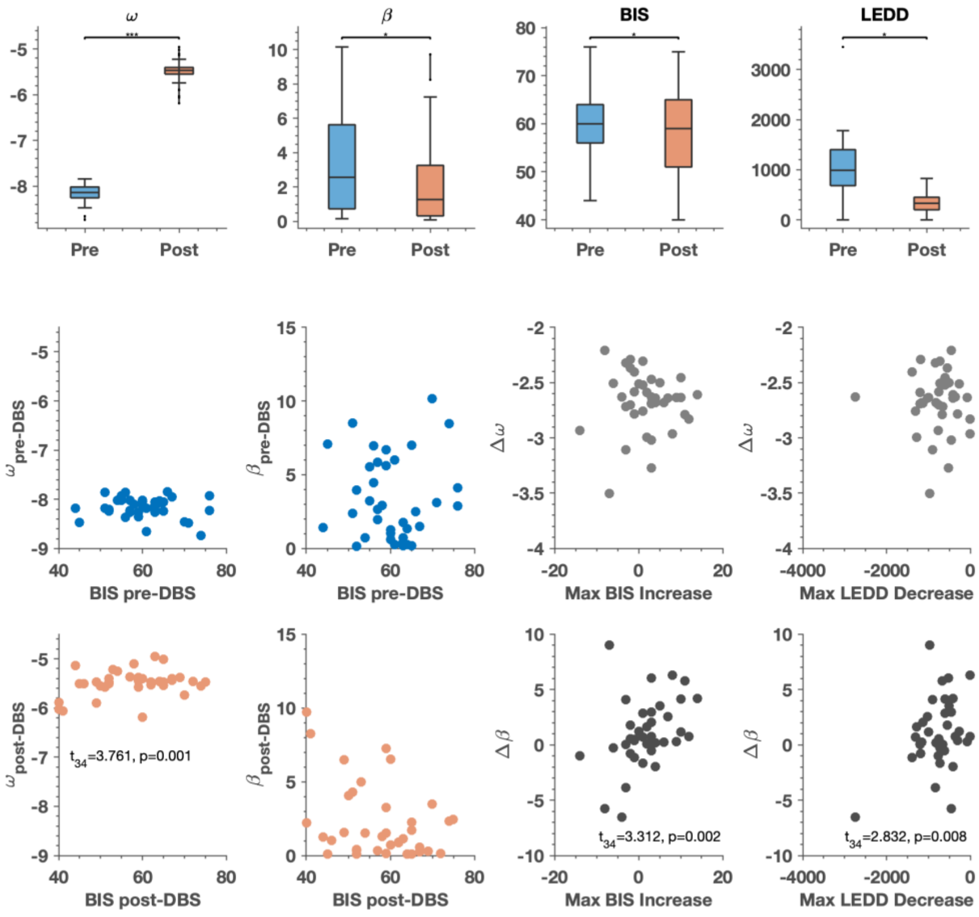

Supplementary Figure 5 | Computational Model Parameters, Pre-and Post DBS. Shown in the figure above is a version of Figure 4 in the main manuscript, with one change. Here, we have included the outlier in assessing the relationship between the pre-to-post operative change in β with the Max LEDD Decrease. As can be seen here, the outlier is driving the statistical relationship between these variables. The full figure is replicated here to provide the context of these analyses.

1.12 Gambling Behaviour

**Supplementary Table 3 | Gambling Behaviour, Pre- and Post-DBS**			
	**Pre-DBS**	**Post-DBS**	**Pre- vs. Post-DBS**
	Mean (SD), Median (Range)		*t-stat (p-value)*
Bet Increases	39.732 (±42.98; 5-174.4)	54.896(±69.261; 5-339.75)	-1.873 (0.069)
Machine Switches	2.052 (±2.968; 0-12)	1.079 (±1.978; 0-10)	1.837 (0.074)
Cashout	0.136 (±0.342; 0-1)	0 (±0; 0-0)	--
Double-up	10.736 (±9.841; 0-25)	13.132 (±9.510; 0-25)	-1.483 (0.147)

Group means are reported with standard deviations and range in parentheses. Notably, subjects elected not to engage in the 'cashout' option post-DBS; this variable was eliminated in subsequent behavioural analyses. Paired t-tests were used to assess the difference in pre- and post-DBS behaviours. No behaviour is significantly different across intervals.

1.13 Pre-DBS Regression of Gambling Behaviour with BIS Subscales

Supplementary Table 4 | Pre-DBS Slot Machine Behaviour and Pre-DBS BIS Subscales

	**Independent Variables (*b*)**						
**Dependent Variables**	**Bet Size**	**Machine Switch**	**Double-up**	**BDI**	R^2	**F-stat**	***p*-value**
BIS-11 Non-Planning	0.012	-0.028	-0.056	0.343	0.179	1.801	0.152
BIS-11 Motor	-0.003	0.266	-0.046	0.138	0.094	0.858	0.499
BIS-11 Attentional	0.025^	0.100	0.025	3.314	0.332	4.094	0.008**

b values are standardized regression coefficients. ****p*<0.001, ***p*<0.01, **p*<0.05 *where p-values are Holm-Bonferroni corrected for multiple comparisons with α = 0.05. ^ Indicates significant t-statistics, Holm-Bonferroni corrected for multiple comparisons.*

1.14 Post-DBS Regression of Gambling Behaviour with BIS Subscales

Supplementary Table 5 | Post-DBS Slot Machine Behaviour and Post-DBS BIS Subscales

	**Independent Variables**						
**Dependent Variables**	**Bet Size**	**Machine Switch**	**Double-up**	**BDI**	R^2	**F-stat**	***p*-value**
BIS-11 Non-Planning	0.006	-0.237	0.031	0.439	0.303	3.588	0.016*
BIS-11 Motor	-0.004	0.467	0.115	0.026	0.250	2.753	0.044*
BIS-11 Attentional	0.016^	-0.101	0.123^	0.254	0.496	8.123	<0.001***

b values are standardized regression coefficients. ****p*<0.001, ***p*<0.01, **p*<0.05 *where p-values are Holm-Bonferroni corrected for multiple comparisons with α = 0.05. ^ Indicates significant t-statistics, Holm-Bonferroni corrected for multiple comparisons.*

1.15 Post-DBS Regression of Gambling Behaviour with QUIP-RS

The QUIP and LEDD correlated strongly at both time points (ρ_{pre}=0.42, p=0.008; ρ_{post}=0.44, p=0.005), with LEDD decreasing significantly post-DBS. LEDD was therefore included as a covariate when regressing against QUIP-RS total scores, in order to explain the remaining variance in QUIP due to gambling behaviour. This is consistent with a previous association of dopaminergic medication with compulsive behavioural disorders in PD.[12]

Supplementary Table 6 presents the relationship between post-DBS gambling behaviour and post-DBS impulsive and compulsive behaviour as measured by the QUIP. Higher bets ($t_{(37)}$=2.057, p=0.048) and more frequent machine switches ($t_{(37)}$=3.268, p=0.016) correspond with higher QUIP scores.

Supplementary Table 6 | Post-DBS Gambling Behaviour and post-DBS QUIP

	**Independent Variables (*b*)**						
**Dependent Variables**	**Bet Size**	**Machine Switch**	**Gamble**	**LEDD**	R^2	**F-stat**	***p*-value**
QUIP	0.073^	3.268^	-0.137	0.004	0.248	2.727	0.046*

*b values are standardized regression coefficients. *p<0.05. ^ Indicates significant t-statistics, Holm-Bonferroni corrected for multiple comparisons.*

1.16 Pre-DBS Regression of Model parameters with BIS Subscales

Supplementary Table 7 | Pre-DBS Model Parameters and Pre-DBS BIS Subscales

Dependent Variables	Independent Variables			R^2	F-stat	*p*-value
	ω	β	BDI			
BIS-11 Non-Planning	-3.268	-0.056	0.326	0.172	2.351	0.090
BIS-11 Motor	-1.222	-0.088	0.085	0.020	0.226	0.877
BIS-11 Attentional	-0.817	-0.225	0.318	0.226	3.314	0.031*

b values are standardized regression coefficients. ****p*<0.001, ***p*<0.01, **p*<0.05 *where p-values are Holm-Bonferroni corrected for multiple comparisons with α = 0.05. ^ Indicates significant t-statistics, Holm-Bonferroni corrected for multiple comparisons.*

1.17 Post-DBS Regression of Model parameters with BIS Subscales

Supplementary Table 8 | Post-DBS Model Parameters and Pre-DBS BIS Subscales

Dependent Variables	Independent Variables			R^2	F-stat	*p*-value
	ω	β	BDI			
BIS-11 Non-Planning	7.024^	0.142	0.480	0.403	7.642	<0.001***
BIS-11 Motor	5.259	-0.022	0.139	0.175	2.400	0.085
BIS-11 Attentional	5.150^	-0.127	0.324	0.407	7.777	<0.001***

b values are standardized regression coefficients. ****p*<0.001, ***p*<0.01, **p*<0.05 *where p-values are Holm-Bonferroni corrected for multiple comparisons with α = 0.05. ^ Indicates significant t-statistics, Holm-Bonferroni corrected for multiple comparisons.*

2. References:

1 Isaias, I. U. *et al.* The relationship between impulsivity and impulse control disorders in Parkinson's disease. *Movement disorders* **23**, 411-415, doi:10.1002/mds.21872 (2008).

2 Voon, V. *et al.* Factors associated with dopaminergic drug-related pathological gambling in Parkinson disease. *Archives of Neurology* **64**, 212-216, doi:10.1001/archneur.64.2.212 (2007).

3 Obeso, I. *et al.* Deficits in inhibitory control and conflict resolution on cognitive and motor tasks in Parkinson's disease. *Exp Brain Res* **212**, 371-384, doi:10.1007/s00221-011-2736-6 (2011).

4 O'Callaghan, C., Naismith, S. L., Hodges, J. R., Lewis, S. J. & Hornberger, M. Fronto-striatal atrophy correlates of inhibitory dysfunction in Parkinson's disease versus behavioural variant frontotemporal dementia. *Cortex; a journal devoted to the study of the nervous system and behavior* **49**, 1833-1843, doi:10.1016/j.cortex.2012.12.003 (2013).

5 O'Callaghan, C. *et al.* A novel bedside task to tap inhibitory dysfunction and fronto-striatal atrophy in Parkinson's disease. *Parkinsonism & related disorders* **19**, 827-830, doi:10.1016/j.parkreldis.2013.04.020 (2013).

6 Milenkova, M. *et al.* Intertemporal choice in Parkinson's disease. *Movement disorders* **26**, 2004-2010, doi:10.1002/mds.23756 (2011).

7 Housden, C. R., O'Sullivan, S. S., Joyce, E. M., Lees, A. J. & Roiser, J. P. Intact reward learning but elevated delay discounting in Parkinson's disease patients with impulsive-compulsive spectrum behaviors. *Neuropsychopharmacology* **35**, 2155-2164, doi:10.1038/npp.2010.84 (2010).

8 Voon, V. *et al.* Impulse control disorders in Parkinson disease: a multicenter case--control study. *Annals of neurology* **69**, 986-996, doi:10.1002/ana.22356 (2011).

9 Paliwal, S., Petzschner, F. H., Schmitz, A. K., Tittgemeyer, M. & Stephan, K. E. A model-based analysis of impulsivity using a slot-machine gambling paradigm. *Frontiers in human neuroscience* **8**, 428, doi:10.3389/fnhum.2014.00428 (2014).

10 Whiteside, S. P. & Lynam, D. R. The Five Factor Model and impulsivity: using a structural model of personality to understand impulsivity. *Personality and Individual Differences* **30**, 669-689, doi:10.1016/S0191-8869(00)00064-7 (2001).

11 Mathys, C. D. *et al.* Uncertainty in perception and the Hierarchical Gaussian Filter. *Frontiers in human neuroscience* **8**, 825, doi:10.3389/fnhum.2014.00825 (2014).

12 Weintraub, D. *et al.* Impulse control disorders in Parkinson disease: a cross-sectional study of 3090 patients. *Archives of Neurology* **67**, 589-595, doi:10.1001/archneurol.2010.65 (2010).
